# Developing a Guided Web App for Postpartum Depression Symptoms: User-Centered Design Approach

**DOI:** 10.2196/56319

**Published:** 2024-08-19

**Authors:** Pamela Franco, Marcia Olhaberry, Antonia Muzard, Ángeles Harismendy, Saskia Kelders

**Affiliations:** 1 School of Psychology Social Sciences Faculty Pontificia Universidad Católica de Chile Santiago Chile; 2 Millennium Institute for Research in Depression and Personality Santiago Chile; 3 School of Psychology Finis Terrae University Santiago Chile; 4 Centre for eHealth & Well-being Research Department of Psychology, Health & Technology University of Twente Enschede Netherlands; 5 Optentia Research Unit North-West University Vanderbijlpark South Africa

**Keywords:** internet-based intervention, postpartum depression, user-centered development, perinatal mental health, user-centered design, mobile phone

## Abstract

**Background:**

Psychological internet-based interventions have shown promise in preventing and treating perinatal depression, but their effectiveness can be hindered by low user engagement. This challenge often arises from a misalignment between technology attributes, user needs, and context. A user-centered, iterative approach involving all stakeholders is recommended.

**Objective:**

In this paper, we aimed to develop a user-friendly psychological internet-based intervention aimed at addressing the symptoms of perinatal depression through an iterative, user-centered approach.

**Methods:**

The development process followed the Center for eHealth Research and Disease Management Roadmap phases of contextual inquiry, value specification, and design. It involved a comprehensive literature review, 2 surveys, 10 focus groups, 5 usability interviews, and 1 technical pilot.

**Results:**

The contextual inquiry revealed a demand for accessible interventions for perinatal mental health, with internet-based solutions seen as viable options. Insights from the literature influenced intervention content and features. Stakeholders’ openness to the intervention became evident during this phase, along with the integration of the first set of values. Initially, we assessed the broader perinatal context to identify the optimal period for the intervention. On the basis of the findings and practical considerations, we decided to specifically target postpartum depression symptoms. The value specification phase further defined the central values and translated them into requirements. In the design phase, feedback was obtained on the user experience of an early digital prototype and on the prototype’s final version. The resulting intervention, named Mamá, te entiendo (“Mom, I get you”), is a guided web app based on cognitive behavioral therapy principles, integrating elements from attachment and mentalization theories. It aims to reduce depressive symptoms in women during the first months postpartum and consists of 6 core sequential modules, along with 3 additional modules, including 5 case examples illustrating depressive symptoms and therapeutic techniques. The intervention provides homework exercises and offers users the opportunity to receive feedback from an e-coach through the web app.

**Conclusions:**

This study emphasizes the importance of a user-centered and iterative development process for psychological internet-based interventions. This process helps clarify user needs and provides valuable feedback on service design and quality, ultimately having the potential to enhance the utility and, presumably, the effectiveness of the intervention. The Discussion section shares valuable insights from the project, such as the value of the requirement sessions.

## Introduction

### Background

Information and communication technologies offer a significant opportunity to overcome logistical and economic challenges in mitigating the burden of perinatal depression [1]. Most existing psychological internet-based interventions (IBIs) for the prevention and treatment of perinatal depression incorporate evidence-based techniques, such as cognitive behavioral therapy (CBT). Despite promising outcomes, these interventions often face high attrition rates, a problem persisting even with the inclusion of human support [2]. Addressing attrition is crucial, as it significantly impacts the overall effectiveness of the interventions [3]. The tendency to overly rely on theory- or expert-driven development methods without sufficient user involvement is a potential factor contributing to higher attrition and lower adoption rates [4]. Early and integrative inclusion of user involvement in the development process has been suggested as a strategy to address these challenges [1,4,5]. The Center for eHealth Research and Disease Management (CeHRes) Roadmap, developed by van Gemert-Pijnen et al [4], offers a guideline for eHealth development, combining evidence-based methods from different disciplines and approaches, such as user-centered design and persuasive technology. It specifically emphasizes the importance of integrating user feedback throughout the development process, ensuring that the intervention aligns closely with user needs and preferences, thereby potentially improving adherence and reducing attrition rates.

This study focuses on developing a psychological IBI aimed at addressing symptoms of perinatal depression through an iterative, user-centered approach. Initially, we evaluated the broader perinatal context to determine the most suitable period for intervention. On the basis of the exploration results and practical considerations, we decided to specifically target postpartum depression symptoms. The study aligns its objectives with the first 3 phases of the CeHRes Roadmap: contextual inquiry, value specification, and design. The overarching goal was to create a user-friendly system that reflects the values of potential users and other stakeholders. In the contextual inquiry phase, we aimed to explore the existing knowledge about IBIs for perinatal depression and determine specific issues where the IBI can add value in our context. This phase established the initial set of values and requirements. During the value specification phase, the focus shifted to further detailing these identified issues and translating the values into specific technological requirements. This phase involved understanding the needs of key stakeholders for an IBI to address postpartum depression symptoms and determining the main values and features that should be included in such an intervention. The outcomes of the value specification phase served as a comprehensive blueprint for the technology development in the design phase. This final phase involved developing the prototype, integrating persuasive elements to enhance user engagement, and conducting usability tests with potential users. We explored whether an early digital prototype and the prototype’s final version met the needs and wishes of potential users and gathered their suggestions for possible improvements.

### Objective

Thus, the user-centered approach of this study, guided by the CeHRes Roadmap, inherently aims to enhance adherence by ensuring that the intervention aligns closely with user needs and preferences, fostering sustained engagement and potentially increasing its effectiveness. The project management team comprised researchers in perinatal mental health and eHealth technologies, clinical psychologists specializing in perinatal mental health, web designers, and software developers. In addition, the feasibility and acceptability of this intervention have already been assessed through a small-scale randomized controlled trial, the results of which have recently been published [6]. We are currently planning a large-scale trial to evaluate intervention efficacy.

## Methods

### Ethical Considerations

The Health Sciences Ethical Committee of Pontificia Universidad Católica (Santiago, Chile; protocol ID 210824004) approved all the development process methods. Informed consent was required for participation in each research activity. The development process took place from February 2020 to February 2023.

### Contextual Inquiry

In this phase, we aimed to gain a better understanding of the Chilean context regarding perinatal mental health; the international evidence on IBIs for depression, specifically perinatal depression; and the perspectives and opinions of perinatal women and health professionals in Chile on perinatal apps. This comprehensive exploration enabled us to create our initial list of values and requirements.

#### Literature Scan

We conducted desk research to gain a deeper understanding of perinatal mental health within the broader Chilean context, as well as international evidence on IBIs for depression, particularly perinatal depression. In addition, we performed a systematic review, which aimed to explore the range and characteristics of psychological IBIs designed to prevent or treat perinatal depression, as documented in randomized controlled trials involving adult women.

The systematic search was conducted in 2020 in CENTRAL, Embase, PsycINFO, and PubMed databases using a combination of perinatal, depression, and internet terms in English. Article abstracts were screened for eligibility by 2 researchers, and eligible papers were retrieved for full-text screening. In developing the intervention, our objectives encompassed gaining insights into the effectiveness of the interventions assessed in the studies included in the review. We aimed to discern the therapeutic approach or theory underpinning these interventions, ascertain the content they covered, determine their length and the presence of modular components (if applicable), evaluate whether they involved human support, and review the adherence to the intended use of the intervention.

#### Web-Based Survey

In addition, we conducted a survey in this phase aimed at assessing views toward pregnancy and parenting apps among perinatal women and health care professionals in Chile. In perinatal women, we explored pregnancy and parenting app use, what they value in the apps they use, and what they considered an “ideal app” for supporting them during this period. We explored health professionals’ opinions on perinatal apps used by women and what they believed a perfect app for their clients should entail. The survey was conducted through a secure web-based platform between September 2020 and October 2020. Participants were recruited through posts and advertisements on social media. Further details regarding this survey can be found in a separate paper [7]. The survey results were thoroughly discussed with the development team, with a particular focus on the information concerning perinatal mental health and user preferences for app features.

### Value Specification

The value specification phase aimed to explore the need for an IBI for perinatal depression and further elaborate on the main values and features that should be included in such an intervention. It involved conducting 8 web-based focus groups to explore the need for a psychological IBI for perinatal depression symptoms and to identify central values, attributes, and features that should be included in such an intervention. We conducted the focus groups with (1) pregnant women, (2) postpartum women, (3) pregnant couples, (4) postpartum couples, (5) pregnant women and perinatal health professionals, (6) postpartum women, (7) women who had undergone treatment and remission for postpartum depression, and (8) perinatal health professionals. Initially, we planned to conduct 7 groups, with 2 mixed groups involving perinatal women and health professionals (groups 5 and 6). However, after observing the dominance of health professionals in the discussions of group 5, which overshadowed the perspectives of pregnant women, we adjusted our approach for group 6. As participants were already recruited and interested in contributing, we chose not to cancel group 6 but instead conducted it exclusively with postpartum women to ensure their views were adequately represented. To still gather insights from health professionals without influencing the women’s responses, an additional exclusive focus group (group 8) was formed. These adjustments were made to optimize stakeholder engagement and data quality.

We aimed for 5 or 6 participants for each group. For focus groups 1 to 6, the inclusion criteria were as follows: perinatal women must be residing in Chile and being pregnant or having a baby aged 0 to 12 months (depending on the focus group). For focus groups 3 and 4, we asked women to attend with their partners. For focus group 7, the inclusion criteria were as follows: women who were residing in Chile, had a baby in the last 3 years, received a diagnosis and treatment for postpartum depression, and were discharged from therapy due to symptom remission at least 6 months ago. The inclusion criteria for health professionals were that they must be residing in Chile and being involved in the care of perinatal women. We provided the following examples in the recruiting flyer: psychologists or psychiatrists specializing in perinatal mental health, midwives, gynecologists, and doulas. All participants were recruited through posts and advertisements on social media.

The first 6 focus groups followed the same structure. After a brief introduction to the research project, we described what psychological IBIs are (as there are no psychological IBIs for adults in the country) and what they might entail. Participants were then asked for their thoughts on accessing an IBI to enhance their psychological well-being and address depression symptoms during the perinatal period. They were also asked to provide input on the characteristics and features they would like to see in the intervention. The focus group with women who had undergone treatment and remission for postpartum depression involved questions about their experience with face-to-face psychological treatment and what characteristics and features they would like to see in an IBI and the e-coaching. The focus group, consisting solely of perinatal health care professionals, also aimed to create descriptions of 5 “typical” Chilean mothers to exemplify the techniques taught throughout the intervention. At the end of each focus group, a book (illustrated about characteristic pregnancy moments) was raffled among the participants.

The 8 focus groups were transcribed and analyzed by 2 independent coders (one of whom is the first author, and the other is a researcher independent of the project). Inductive content analysis was used to identify response patterns [8]. The second and third authors reviewed the categories and subcategories map and provided feedback. The information collected was summarized based on the main themes and discussed with the project development team in requirement sessions (refer to Multimedia Appendix 1 for the focus groups script).

### Design Phase

#### Overview

In this phase, we explored whether certain features of an early digital prototype and its final version met the needs and preferences of potential users. We assessed its user-friendliness and functionality and gathered suggestions for possible improvements. For this purpose, we conducted 2 focus groups, 5 usability interviews (UIs), and a technical pilot (TP). The inclusion criteria comprised women residing in Chile with babies aged 1 to 6 months. The criterion for the baby’s age was determined based on the findings from the previous phase (mentioned in the Results section). Participants were recruited through posts and advertisements on social media.

#### Focus Groups

During the focus groups, we presented a set of photographs and illustrations for the prototype to gather feedback on general impressions, represented situations, representativeness of Chilean mothers, and emotional tone. We also presented screenshots of a “Workbook” section (with therapeutic exercises) to assess the value of having this function and gathering feedback on the design. Subsequently, participants were asked to explore the prototype on their smartphones and provided feedback on its sections, user-friendliness, and design. We also assessed whether participants found it feasible to complete 1 module per week. Finally, participants were presented with a list of themes for developing infographics and asked to rate their relevance on a scale from 1 (not relevant) to 5 (very relevant).

For the focus groups, the following materials were prepared: (1) a set of photographs selected from an open database, (2) one developed illustration for each 1 of 5 example cases of mothers, (3) screenshots of the Workbook, and (4) a high-fidelity clickable short version of the prototype of the intervention. The prototype was developed on the Figma website (Figma [9]) and included the registration procedure, the first 2 intervention modules (Introductory module and Psychoeducation on postpartum depression), 1 infographic, the reading for the support network, and the Workbook section. The illustrations, the photographs, and the overview of the Workbook are presented in Multimedia Appendix 2.

The head of designers, who was present in the 2 focus groups from the design phase, took notes along with the interviewer. After each focus group, a debriefing meeting was held between the interviewer and the designer. The focus groups were transcribed and analyzed using inductive content analysis [8]. The information collected was summarized based on the main themes and discussed with the project development team in requirement sessions (refer to Multimedia Appendix 1 for the focus groups script).

#### UIs: Think Aloud Method

An extended version of the clickable prototype, encompassing all the modules and sections of the platform, was developed for the UIs. Participants accessed the prototype on their smartphones while using the think aloud method [10]. We encouraged participants to navigate the prototype on their smartphones and speak their thoughts aloud as they explored it. The interviewer’s questions primarily served as prompts to keep them talking and gain a deeper understanding of any points raised by the participants.

Given the extensive amount of information in the prototype, we instructed participants not to concentrate on the content but on aspects such as design, user-friendliness, and features. Toward the conclusion of the interview, participants were asked 3 questions: (1) “On a scale from 1 to 10, how would you rate the user-friendliness of the prototype, and why?” (with 1 indicating “not user-friendly” and 10 signifying “very user-friendly”), (2) “Would you consider using this intervention if you were experiencing low mood? If so, why?” and (3) “Would you recommend this intervention to a friend with low postpartum mood? Please explain your rationale” (refer to Multimedia Appendix 1 for the interview script).

The interviews were transcribed, and all comments were categorized into 2 groups: positive aspects highlighted by the users concerning the prototype and suggestions for improvement. These categorized comments were then organized into main themes and discussed during the requirements sessions.

#### TP Phase

After developing the technology, a small TP was conducted to ensure the functionality and reliability of the web app. This step is crucial to identify and address any programming flaws before proceeding with the feasibility and acceptability study. Ensuring that the app operates smoothly is essential to maintaining the integrity of the research and providing a reliable user experience. Given that the primary goal of this pilot was not to assess feasibility, acceptability, or effectiveness, it was not necessary to statistically calculate the sample size for this phase. Instead, we aimed to recruit a practical number of participants to allow for adequate testing of the technology’s functionality. Consequently, 10 women with infants aged between 1 and 6 months were recruited. Having symptoms of depression was not an inclusion criterion; however, we requested participants to complete the Chilean version of the Edinburgh Postpartum Depression Scale (Edinburgh Postnatal Depression Scale [EPDS]; 10 items, range 0-30; cutoff values of ≥10 identify women who might have depression) [11] before accessing the technology to characterize the sample. We also documented whether the participant was presently receiving mental health treatment. Recruitment was conducted using a convenience sampling approach, asking researchers and psychotherapists not affiliated with the study to refer potential participants.

Throughout this pilot phase, participants were granted full access to the technology, including e-coach support, for 6 weeks, enabling them to explore all its features. The primary objective was ensuring the functionality and reliability of the technology rather than collecting data on its impact or user opinions. Our main focus was to gather information related to log data and identify potential bugs (ie, coding errors) or issues. If participants encountered a bug or a significant usability issue, they promptly reported it to the study coordinator, who relayed this information to the software developers for timely resolution. We kept records of the number of completed modules and exercises submitted and tracked the identification and nature of any issues discovered.

As a secondary objective, we assessed satisfaction and engagement with the technology. Upon the conclusion of the 6 weeks, participants were asked to complete 2 assessment tools: the Spanish version of the Client Satisfaction Questionnaire-8 (CSQ-8; 8 items) [12] and a Spanish version (using back and forward translation) of the Twente Engagement with eHealth Technologies Scale (TWEETS; 9 items) [13]. Descriptive analyses were conducted to analyze the scores obtained from the CSQ-8 and TWEETS.

### Formative Evaluation: Requirement Sessions

After each research method, the research team (comprising researchers in psychotherapy who are also psychotherapists and researchers in eHealth) discussed the results. The results were then communicated to the design and development team through reports written by the researchers, and those reports were discussed with them in several meetings. The results were translated into requirements by the designers and software developers, and the researchers verified whether the requirements met the expected needs.

## Results

### Contextual Inquiry

#### Literature Scan

In Chile, perinatal women attending public health facilities are universally screened for depression symptoms by the EPDS during the second pregnancy check-up and the baby’s 2- and 6-month checks [14]. If there is a suspicion of depression diagnosis, they are referred for further evaluation. In the country, adults diagnosed with depression have guaranteed access to treatment at low or no cost, which includes psychotherapy, pharmacotherapy, or both [15]. Despite these measures, perinatal depression prevalence in Chile remains high, and it is often undertreated [16]. The barriers for perinatal women seeking mental health care include misconceptions about mental disorders and psychotherapy, fear of being judged as a “bad mother,” lack of time or energy, and difficulties attending health care centers in person [17]. Providers’ barriers include a lack of training on mental health for perinatal health care professionals [17], long waiting lists for mental health services, frequency and duration of sessions below international recommendations, and high staff turnover [18].

Moreover, there is a lack of interventions in the country for perinatal women exhibiting depressive symptoms who do not meet the criteria for a major depression diagnosis, such as subthreshold depression. Subthreshold depression is more prevalent than major depression, making its associated costs comparable, and has a significant impact on quality of life and health status, with an increased risk of developing major depression [19,20]. Psychological interventions aimed at reducing subthreshold depression symptoms and preventing the onset of major depression [21] could also help reduce the treatment gap. By contrast, studies in the country have reported a high prevalence of anxiety symptoms among perinatal women, with about 40% of women presenting moderate to severe symptoms of anxiety according to the Perinatal Anxiety Screening Scale [22]. This finding is significant, as evidence indicates that comorbid perinatal anxiety and depression are common and associated with greater symptom severity [23].

Regarding the systematic review of psychological IBIs for preventing and treating perinatal depression, only the most relevant information for the intervention development is presented here, with detailed conclusions published elsewhere [24]. The systematic review identified 15 effectiveness studies on interventions for perinatal depression [25-39]. The majority were conducted in high-income countries. We identified a multicenter study conducted by a research team in the United States involving participants who spoke both Spanish and English and included individuals from 23 countries, including South American nations [27]. However, we could not identify a study specifically conducted within South America.

In 11 studies, significant differences favoring the intervention were observed in depression symptoms in the intervention group compared with the control group, which predominantly consisted of treatment as usual and, in a minority of cases, a waitlist group. Most of the interventions used a CBT approach. Approximately half of the interventions included some form of human guidance or support, typically delivered by a clinical psychologist. The interventions were usually structured into sequential modules meant to be completed weekly. Most incorporated multimedia-presented therapeutic content and homework assignments for practicing the acquired skills. Case examples were frequently integrated to illustrate therapeutic concepts. Most studies identified in the systematic review that assessed intervention acceptability showed good participant satisfaction levels. However, many studies reported a high rate of intervention attrition.

The literature scan showed some characteristics that might enhance the effectiveness of an IBI: providing some form of human support [3,40,41], ensuring cultural sensitivity [42], incorporating persuasive technology features [4,43,44], and having a 7-module length [3]. By contrast, research on face-to-face treatments for perinatal depression highlights the importance of improving the quality of the mother-infant relationship as a protective factor for both the mother and child, achieved through enhancing maternal sensitivity and the mother-infant bond [45]. Theories rooted in mentalization and attachment play a vital role in interventions for mothers with infants by promoting secure bonding, improving comprehension of the baby’s needs, facilitating emotional regulation, and nurturing healthy infant development [46,47].

Our literature scan also identified self-help books and manuals for perinatal distress, depression, and anxiety.

#### Web-Based Survey

The survey was completed by 451 perinatal women and 54 perinatal health professionals. Here, we provide a summarized overview of the most important findings for the development process; detailed results are published elsewhere [7]. The results indicated that perinatal women in Chile frequently use perinatal apps, and both women and health care professionals hold a positive view of them. The most highly valued aspect of these apps was the information they offer, which mainly targets baby development in the uterus or after birth, week-by-week changes during pregnancy, and guidance on maintaining health throughout pregnancy and postpartum. The survey further unveiled various requisites for an “ideal app.” A visually appealing interface with visuals such as images, illustrations, graphs, or videos, as well as user-friendliness and personalization options, were sought-after features. Content encompassing both pregnancy and postpartum aspects, grounded in evidence or endorsed by health care professionals, and offering relevant and up-to-date tips was deemed crucial. Participants desired the capability to interact with both perinatal health care professionals and other perinatal women in the community.

Addressing women’s mental health emerged as a prominent concern among both perinatal women and health care professionals, an area seemingly underaddressed in the apps they use. Recommendations for mental health care included strategies for mental health promotion, psychoeducation, well-being monitoring through questionnaires, advice on managing perinatal depression and anxiety symptoms, and guidance on seeking professional help.

Both perinatal women and health care professionals expressed a desire for app sections targeting partners. In addition, perinatal women emphasized the importance of the app addressing the couple’s transition to parenthood.

#### Requirements Sessions

At this stage, it was decided that the IBI would primarily serve as a research tool. This decision was motivated by practical considerations, as there was no specific implementation setting for the intervention, such as a participating mental health care organization or institution. The absence of institutional participation also meant that the budget for developing the intervention was very limited. In addition, the project team aimed to experiment with the IBI, and this approach was deemed most feasible as a research tool. Consequently, the most critical stakeholder groups were identified. Given the focus on a research implementation setting, care providers played a less prominent role in the development process during this stage.

Several decisions were reached during the researchers’ meetings, drawing from insights gathered during the contextual inquiry phase. The literature scan on the Chilean context has highlighted several critical needs that our intervention aims to address. First, there is a pressing need for interventions that are easily accessible and flexible, particularly for managing major perinatal depression, to reduce the treatment gap. Second, there is a requirement for interventions designed for the indicated prevention of perinatal depression, which could prevent the progression from subthreshold to major depression. Finally, given the high prevalence and impact of comorbid anxiety symptoms among perinatal women, it is crucial for the intervention also to address anxiety if comorbidity is present. These specifications are essential for developing a comprehensive and practical approach to perinatal mental healthcare. The intervention will be rooted in CBT principles, supplemented with elements from attachment and mentalization theories, and will include homework exercises with personalized feedback provided by an e-coach. The intervention should compass psychoeducation, strategies for promoting mental health and managing perinatal depression and anxiety symptoms, and guidance on seeking professional assistance. In addition, it should include information on how to enhance the couple’s relationship functioning during the transition to parenthood and provide information for partners.

The language, examples, and visual materials must be culturally sensitive. The technology must have a visually appealing interface, be user-friendly, and allow some personalization. Persuasive elements will be incorporated following the persuasive systems design framework [43].

### Value Specification

#### Participants

A total of 39 perinatal women and health care professionals participated in the 8 focus groups. None of the women from the pregnant and postpartum couples’ groups attended with their partners, so these groups were conducted with only women. Perinatal women had an average age of 32.6 (SD 4.6; range 25-42) years. Pregnant women were, on average, at 28.7 (SD 7.3; range 15-39) weeks gestation. The average age of postpartum women’s babies was 4.2 (SD 2.4; range 1-9) months. More than half of the perinatal women (22/31, 71%) were primiparas. Most perinatal health care professionals (6/8, 75%) were psychotherapists specialized in perinatal mental health. Table 1 shows the participants’ characteristics presented by group across all phases.

**Table 1:** Characteristics of focus groups (FGs), usability interviews (UIs), and technical pilot (TP) participants.

Part of the study	Target group	Participants, n (%)	Woman’s age (y), mean (range)	Babies’ age (months or g/W^a^), mean (range)	Perinatal health care professionals’ professions
FG1	Postpartum women	6	31.5 (27-38)	5.1 (2-9)	—^b^
FG 2	Pregnant women+perinatal health care professionals	7	30.0 (25-33)	25.3 (19-30)	4 psychotherapists specialized in perinatal mental health
FG 3	Pregnant women	8	33.4 (26-38)	28.4 (15-35)	—
FG 4	Postpartum couples (only women participated)	3	29.6 (27-34)	3.0 (1-6)	—
FG 5	Pregnant couples (only women participated)	6	36.2 (28-42)	36.2 (34-39)	—
FG 6	Postpartum women	3	31.5 (30-33)	4.5 (3-6)	—
FG 7	Women with treated and remitted postpartum depression	2	34.0 (27-41)	18.0 (12-24)	—
FG 8	Perinatal health care professionals	4	—	—	2 psychotherapists specialized in perinatal mental health, 1 gynecologist, 1 social worker, 1 lactation consultant, and 1 doula
FG 9	Postpartum women	5	35.5 (30-43)	3.8 (1-6)	—
FG 10	Postpartum women	5	32 (25-40)	3.0 (1-5)	—
UI	Postpartum women	5	33.6 (26-39)	5.2 (4-7)	—
TP	Postpartum women	10	34.5 (31-40)	3.8 (2-5)	—

^a^g/W: gestational weeks.

^b^Not applicable.

#### Participants’ Expected Needs

Table 2 shows the perceived value of an IBI for perinatal mental health and the desired characteristics and features from the participants’ perspective, as derived from focus groups 1 to 7, with quotes to exemplify each category.

In focus group 8 with perinatal health professionals, the discussion explored the most frequent problems or challenges and depression or anxiety symptoms reported by postpartum women in consultations. The debate served as input to create the intervention’s fictitious characters (eg, the “diligent mother” with perfectionist traits and high self-expectations who invested significant effort in educating herself extensively about infant care during pregnancy but then experienced frustration when she realized that motherhood does not unfold as she initially anticipated).

**Table 2:** Results of the exploration of the need for an internet-based intervention for perinatal mental health and the central values and features that should be included in such intervention.

Categories and subcategories				Quote to exemplify the category and subcategory
**Perceived value of such intervention**				
	**It could overcome health care providers’ barriers to care**			
		From public health facilities: long waitlists for mental health evaluation and treatment	“I really think it would be something very, very good, because I don’t think I’m the only one who has encountered problems at the healthcare center where you have to wait 3 or 4 months for them to give you an appointment in mental health care.” [FG1^a^]	
		From private health facilities: low attention to PMH^b^ from perinatal health professionals	“You have to have very specific symptoms to get a referral, or you have to seek help yourself, but there’s no checking to see if you’re experiencing depression and need help. So, I believe this is important because many times one doesn’t know...” [FG3] “I also had my pregnancy and postpartum check-ups at a private clinic, and nobody has ever asked me about my mental health… they didn’t talk to me about postpartum depression either.” [FG6]	
	**It could overcome women’s barriers to care**			
		Less time and effort are required for an internet-based intervention compared with attending in-person treatment	“I would love it because the biggest challenge is time. I’ve talked to many moms, and it has been quite difficult for them to find the time to attend sessions with psychologists, to sit down and talk for that long. Sometimes they have more specific doubts, and generally, as moms, or at least for me, I have more time at night, like when the baby falls asleep; I can take advantage of that time, or sometimes when I’m breastfeeding, or the baby is napping. So, it would be wonderful to have an app to use during those moments, for me, for my mental health.” [FG1] “You have so little time, or you don’t have someone to leave your baby with; they’re still very little, as if to say, ‘I’m going for a psychological evaluation.’” [FG6]	
		Easy access: women already use perinatal apps	“It is a great idea for any woman who is about to become a mom or already is one; Personally, I’m very attached to social media and apps; I downloaded all kinds of apps even before I got pregnant, like period trackers, and then during pregnancy, I used apps to track my baby’s development. So, I’m always looking for these things whenever I have some free time; For example, when the baby is taking a nap or while doing other tasks, I often use my phone for that.” [FG1]	
	“For moms”: participants value the idea of having a virtual space “for them” (the perinatal apps they use focus mainly on the baby)		“[W]hat I find great is that it is focused on the mother, while many of the apps are more focused on the unborn baby, the weeks, the care, and the forums on those apps focus on things like what ultrasound I need to have if I’m at a certain number of weeks. So, everything is focused on the baby, which obviously reduces the mother’s anxiety. However, I hadn’t seen something focused on the mother’s mental health, and nowadays, it’s really necessary.” [FG2]	
	“Made in Chile”: participants value the idea of having a platform made for the Chilean context (the perinatal apps they use are not Chilean)		“I really think it’s very necessary and wonderful to have a Chilean app. Because I also used the same apps as others mentioned, but sometimes they talk about different places in the world, which is great, but sometimes the local context matters too. Like, the cultural aspect...” [FG5]	
	**Perinatal mental health is perceived as an overlooked area that needs care**			
		The perinatal period is perceived as a vulnerable moment for the onset of mental health disorders	“[T]hat stage [puerperium period] is super critical, I think it needs a lot of emphasis because, honestly, I saw her [a friend] in a bad state, and I got really scared; I told her, ‘You have to go to a psychologist,’.... and truly, I feel that since she started therapy, even her relationship with her baby improved. So, I believe that stage is the most critical, and I think it’s the one that scares us the most; I’m also very afraid of that stage.” [FG3] “My son was super planned and super expected, and it is incredible that even if you have it all planned, your day-to-day suffers an abrupt change, and you have to cope with that.” [FG1]	
		Women fear being judged for experiencing distress in the perinatal period	“Motherhood is often experienced in isolation, and you are expected to be happy and joyful because you are pregnant and becoming a mother. So, having a space where we can acknowledge and release those emotions, which might not align with societal expectations of a pregnant woman, is incredibly liberating.” [FG2] “In Chile, the rate of depression is very high, so it makes us feel supported knowing that we are not alone, that we are not the only ones who have it, that we should not be ashamed at all...” [FG3]	
		There is a need to know when they should look for professional care and where	“I was coming out of a depression, and she [psychologist] explained everything to me. She told me, look, what are the signs of postpartum depression, what is normal, so maybe on this page, on this app, I don’t know if a questionnaire would be appropriate, but tips, like, be aware that when you cry suddenly when you see your child, that’s normal, but if you cry all day, every day, for I don’t know how many days, that’s not normal anymore. So, be aware, ask for help, get advice, it’s also normal to ask for help...” [FG3]	
		There is a need for mental health promotion and prevention	“I think it’s something good that would maybe help prevent actually having depression because, I don’t know how to say it in psychological terms, but I believe that if you take care of it long before, prevent postpartum depression, take care of your mental health, and that’s why if there are tools, advice, a place with opportunities to talk, complain, having that space would be great.” [FG2] “Your changes dramatically, so I think the preventive approach is super fundamental.” [FG3]	
		Websites and social media display contradictory information, or they do not know if it is trustworthy	“[S]o, you start to question yourself and think, am I okay? and you start looking for information online, but then you don’t know if it is reliable...” [FG5] “Once I got pregnant, I found out that there was like an underworld of web pages, of information, of Instagram accounts, that when you’re not pregnant, you have no idea, and you get in, and information exists on various levels of reliability.” [FG3]	
**Therapeutic content requests**				
	Psychoeducation: psychoeducation on the transition to motherhood (what is normal or expected and what is not regarding perinatal mental health, how to detect symptoms of depression or anxiety, and when to look for professional help and information about mental health treatment)		“Like I was saying before, everything [other apps] focuses on the intrauterine development of the growing baby and all, but what about us? Who tells us what we are going through emotionally, maternal ambivalence, and the psychic transparency that occurs in the process of pregnancy? No one talks about that. So, understanding that these are natural processes inherent to pregnancy, also allows them to realize and become aware that maybe this pregnancy is a bit more painful, a bit more complex, and that they really need help, or maybe, ah, what I’m experiencing is just part of it, it’s normal. So basically, it’s like having an app, or a platform, or a website that guides you, accompanies you...” [FG2; Perinatal mental health professional] “[A] space for guidance and accompaniment, more than anything, so that a woman knows what her psychological processes are, that also allows her to understand and realize that perhaps what she is feeling, what is happening to her, if that is a little out of line with what it should be within this range, then from there like, like a little alert flag.” [FG2; Perinatal mental health professional]	
	Strategies for dealing with negative emotions: dealing with distress, depression, anxiety, and guilt. Coping with mixed feelings toward motherhood		“Oh, the guilt. When I think, ‘Oh, I love my son, but I don’t want him to cry anymore; Oh, I love my son, but I want him to go to sleep; Oh, I love my son, but I don’t want him to wake up at 5 in the morning ‘. And it is this duality that society does not understand because it throws you the label of a bad mother, Oh, what a bad mother; you do not love your baby... But it is not about that; it is about that we are human beings who get tired, exhausted, we have emotions... Guilt is a common issue in motherhood.” [FG7] “I would have liked for someone to tell me that it’s okay to cry, just cry. It’s alright to feel down for a while; it’s fine. But don’t worry; it’s a natural part of the process. You’ll be able to overcome it; it is an adaptation process.” [FG4]	
	How to empower oneself as a mother: how to develop confidence as a mother, trust own decisions, and worry less about what others think		“I think that one has to learn to filter all that [criticism and unwanted opinions] because if not, it becomes chaos because they criticize you for doing A or for not doing A.” [FG7]	
	How to deal with postpartum body changes		“I couldn’t enjoy sexual relations, and in large part, it was because of the issue with my body. Before, I was very thin, and then I ended up like this. So, of course, in the end, since I didn’t like my own body, how could someone else like it?” [FG4] “I went into a crisis because of my body, a little depressed, but you know, then I was like, ‘Noo! Your body grew a human being inside for nine months, fed it, and you gave birth, you lived what it is to give birth, your body is wonderful.’” [FG7]	
	**Strengthening the support network**			
		The couple’s relationship: to consider the couple’s transition to parenthood, shared parenting, and the partner’s mental health	“Something to make your partner can understand a little of what you are going through and give them tools to support you.” [FG6] “Finally, I realized that many times, my partner didn’t do it, because I didn’t let him either, I didn’t give him the option of making him realize that I needed him to do something.” [FG4] “[B]ecause sometimes they ask, ‘Can I do this?’ and you think, ‘No because you don’t know how to do it like I do’; But the truth is, they know how to do it in their own way; As a couple, we decided to have a child together, and that means we both signed up for this adventure; So, why do I end up carrying the load on my own? I’m not sure if you’ve experienced this, but delegating was tough for me; Not so much anymore, though—now, I’m more comfortable with it.” [FG4] “No one cares about the father; no one cares if he’s tired, sleepy, or hungry... we should also involve the father because he’s a crucial pillar.” [FG4]	
		Any close one: how to ask for help and set boundaries. Shareable information for the support network on how they can support the mother and on perinatal mental health	“Essentially, it’s about informing the person who is closest to you about what you’re going through;... Because you can’t go through this situation alone, you just can’t.” [FG3] “Information for them [close ones]; how can I do it, how do I help a mom with stress, or depression, or who are having a very bad time.” [FG3] “You feel very lonely because you can discuss it [being diagnosed with depression] with your partner, your family, your friends, but it’s like they say, ‘But you’re experiencing one of the best stages, which is carrying your baby. How can you feel down?’.” [FG5]	
**Inclusion of human support**				
	From a therapist: psychological and emotional support and coaching. Homework exercises should be optional		“It is a matter of support, of having someone to talk to, of empathy, because it is clear that it will not solve your economic issues or make the pandemic go away, but one needs empathy... to feel supported without judgment.” [FG7] “Feeling understood and validated.” [FG8] “I think homework should be optional; Sometimes even my therapist gives me homework, and I don’t do it.” [FG5]	
	From other perinatal women: interaction through forums or web-based thematic group workshops embedded in the technology		“It would be beneficial to have opportunities for conversation and support among pregnant women, where we can openly share our struggles and experiences. It sounds interesting and enjoyable to have such a platform where we can connect with and support each other.” [FG2]	
**General requests for an internet-based intervention**				
	To show the “real” motherhood: to deromanticize motherhood and mention the abrupt life changes and the feelings of loneliness. The “perfect mother” does not exist		“[S]o, I believe the app should also include that part, the ugly side, the realistic part of motherhood, because when you have too many expectations, then there is chaos, I feel, and I think knowing that helped me.” [FG3] “It occurred to me that there could also be a section in the intervention with written experiences of mothers, that is, not positive or negative, but real experiences...” [FG3]	
	Preferred timing of intervention: emphasis on early postpartum period		“For me, I think it would be good from the end of pregnancy,... and then when you are at home with the newborn, and he cries, and you get overwhelmed.” [FG4] “I think from birth, more or less, for me, that was the most chaotic moment.” [FG4]	
	**Information characteristics**			
		Short, easy-to-read sections	“I also think about 15 to 20 minutes daily...” [FG1] “Something to read in a short moment, like when I’m breastfeeding, and I have a moment to glance at my phone, alternating between looking at the baby and reading for a bit, or at night when he’s asleep.” [FG4]	
		Evidence-based or reliable	“[I]nstead of searching elsewhere, that’s what’s important, having reliable information.” [FG3]	
	“Relaxing,” “not girly,” “adult” looking		“Perhaps the app should avoid using colors commonly associated with babies by making it evident that it is specifically designed for mothers and not focused on baby care.” [FG1] “Please don’t make it all pink.” [FG5]	

^a^FG: focus group.

^b^PMH: perinatal mental health.

#### Requirements Sessions

The focus groups’ results were discussed initially with the research team and later with the designers. Some categories overlapped with those that emerged in the survey study. The focus groups reinforced the choice to include therapist support. Despite strong demand for support from other perinatal women in both focus groups and surveys, it was not included at this stage, given the intervention’s research focus and the desire to assess its psychotherapeutic components in the future.

Opinions among participants were divided regarding the preferred timing of the intervention during the perinatal period. All pregnant women (17/17, 100%) wanted the intervention to focus on the pregnancy period or both pregnancy and postpartum. Regarding postpartum women, about half (8/14, 57%) preferred the intervention to focus exclusively on the postpartum period, while the rest preferred it to cover both pregnancy and postpartum. When postpartum women (who had recent experience with both periods) were asked to choose only one period, the majority expressed a preference for the postpartum period (13/14, 93%). In addition, postpartum women emphasized the importance of receiving this intervention in the early months following childbirth. Considering these preferences and economical and practical factors and the specific barriers associated with the postpartum period (eg, related to baby care and attending in-person health centers), the decision was made to target the intervention at postpartum women, specifically during the first 6 months after childbirth. During this stage, the intervention modules and sections were defined. The content was divided into short, easy-to-read modules that can be read in 10 to 15 minutes. Modules include providing psychoeducation on depression and anxiety, promoting mental health care help seeking, and providing CBT strategies for dealing with negative emotions. The homework exercises were carefully chosen and adapted from the therapy manual for perinatal depression and anxiety by Green et al [48], which covers the core principles and skills of CBT.

It was decided to include infographics on relevant postpartum topics (such as body changes), a shareable guide on supporting mothers with depression symptoms, an “About” section detailing the development team and funding sources, a “Resources” section with links to maternal and mental health support, and a “Favorites” section for saving preferred content.

The insights from the focus group of perinatal health care professionals informed the creation of fictional characters, who serve as practical examples to illustrate the content and therapeutic techniques. Our design team crafted illustrative representations of these characters in everyday scenarios to ensure a realistic portrayal. To ensure inclusivity for lesbian couples, the gender of the partners of the fictional women was left ambiguous in the text and illustrations. Moreover, we have carefully chosen inclusive language throughout the written content, consistently using “your partner” instead of “the father of the baby.” In addition, one of the female characters in our narratives does not have a romantic partner, reflecting the diversity of family configurations.

Subsequent meetings with designers and software developers focused on refining the interface to ensure it is user-friendly and easy to navigate. It was also defined how elements of persuasive systems design [43] would be applied to the intervention (Table S1 in Multimedia Appendix 3 [44]). Table 3 summarizes the key values, attributes, and requirements for the intervention that emerged from the contextual inquiry and value specification phases.

**Table 3:** Values, attributes, and requirements for an internet-based intervention for reducing postpartum depression symptoms and improving mental health.

Values and attributes	Requirements
The literature scan on the Chilean context revealed a need for the following: A flexible, easily accessible intervention for major perinatal depression to reduce the treatment gap Indicated the prevention of perinatal depression to prevent the escalation from subthreshold to major depression Components to address anxiety symptoms, ensuring comprehensive treatment for women with comorbid conditions	The system targets symptoms of depression, applicable to both diagnosed major depression and subthreshold depression The system includes additional modules on anxiety for comprehensive management in cases of comorbidity
The systematic review and literature scan have indicated that for the system to be engaging and effective, it should possess the following characteristics: Incorporate a CBT^a^ approach Incorporate therapist support Demonstrate cultural sensitivity Include persuasive technology features Foster maternal sensitivity and strengthen the mother-baby bond Distribute the content in approximately 7 modules	The system incorporates 6 core modules and offers 3 optional modules based on the CBT approach The system features an e-coach that provides feedback on optional therapeutic exercises available within the modules Culturally sensitive language, examples, and visual materials are integrated into the system The system includes persuasive technology features, as described in Multimedia Appendix 3 [44] The system’s content includes elements from attachment and mentalization theories
Stakeholders believe that the system should actively promote mental health literacy and care by the following methods: Educating users about perinatal mental health disorders Facilitating the recognition of depression and anxiety symptoms and encouraging help-seeking behavior Equipping users with skills to manage depression and anxiety symptoms and enhance overall well-being Offering symptom questionnaires for self-assessment	A psychoeducational module is included. The CBT modules offer a comprehensive model for understanding depression and anxiety symptoms, along with techniques for managing these symptoms and enhancing well-being, including practice exercises The system contains a “Resources” section with links to websites and hotlines that support mental health Symptom questionnaires will not be included in this version of the system
Stakeholders express a desire for the system’s use during both pregnancy and the postpartum period. A preference is observed among postpartum women for using the system during the initial months post partum	The content and examples within the system are tailored to women with babies in their first 6 months
Stakeholders envision the system as an accurate reflection of “real” motherhood, addressing the most common postpartum challenges and providing guidance on navigating them	The system introduces 5 characters who share their stories and demonstrate therapeutic techniques Infographics addressing common challenges (such as coping with the postpartum body changes) and providing advice on how to cope with them
Stakeholders desire the system to facilitate social interaction, allowing users to connect with therapists and other women experiencing similar situations.	An e-coach is integrated into the system to offer feedback on exercises for women and address any content-related queries Social interaction with other women will not be available in this system version
Stakeholders believe the system should strengthen users’ support networks	The system incorporates a shareable “Reading for Loved Ones” section, providing guidance on supporting a woman experiencing postpartum depression symptoms One of the extra modules centers on enhancing communication skills An infographic addresses the couple’s transition to parenthood and strategies for nurturing the couple’s relationship during the postpartum period
Stakeholders emphasize the importance of a visually appealing interface for the system	The system features pictures depicting scenes related to the postpartum period, illustrations of the 5 characters, and diagrams to facilitate information presentation The system uses a color palette designed to appeal to adult women

^a^CBT: cognitive behavioral therapy.

### Design

#### Participants

A total of 10 women with babies aged 1 to 6 months participated in focus groups 9 and 10, 5 in the UIs, and 10 in the TP. The mean age of the women and their babies was 33.7 (SD 6.3; range 25-43) years and 3.6 (SD 1.4; range 1-6) months, respectively, in the focus groups; 33.6 (SD 6.9; range 26-39) years and 5.2 (SD 1.2; range 4-7) months, respectively, in the UIs; and 34.5 (SD 3.5; range 31-40) years and 3.8 (SD 1.3; range 2-6) months, respectively, in the TP. The majority (16/25, 64%) were primiparas.

In the TP group, women had an average score of 9.60 on the EPDS (SD 5.30; range 3-18); 4 (40%) women were currently undergoing psychological treatment for postpartum depression symptoms. A total of 7 (70%) women completed the CSQ-8 and the TWEETS after the 6-week access period.

#### Focus Groups: Participant Feedback

##### Feedback on the Set of Illustrations

Participants appreciated that the illustrations depicted challenging daily situations faced by mothers. Moreover, 1 participant expressed how the images shattered unrealistic myths about motherhood:

I find that they are very realistic and also tear down those myths of motherhood or pregnancy, of moms always happy and almost making postpartum depression invisible, when in truth, one is very tired, one experiences a lot of emotions, one has little energy.

In both focus groups, 1 participant mentioned wanting the mothers to be more accompanied:

[I]n the end, maternity is quite lonely, but eh, I think that too, but I don’t know, the message could be that I need to be more accompanied, to have more tribe, more family around.

The participants generally agreed that the mothers represented the “Chilean mother visually,” with good diversity among them. While most participants found the facial expressions realistic and relatable, 2 felt that the sole display of overwhelmed expressions was not ideal:

The facial expression came to me; it was the first thing I looked at... “Oh, the afflicted face, it’s me all the time,” I saw myself in all their faces... It does come to me, and I connected with the images, how I am in bed, lying with the baby on top.

I know that motherhood is difficult, but seeing a super sad, super frustrated mom makes me sad because I try to be there for my baby and not be sad.

##### Feedback on the Set of Photographs

Participants appreciated the portrayal of a wide range of emotions, positively valued the esthetics of the photos, and especially praised the depiction of family diversity and coparenting situations in the various photographs:

[H]ere you can see different situations, not only the negative side, but the positive side of motherhood, enjoying her baby or also with friends or with family, or having time for themselves, as writing or meditating.

However, some participants (3/10, 30%) felt that the photographs did not fully represent the diverse profiles of Chilean mothers, as they primarily depicted young and slim women, thereby representing mainly high-income mothers and not the broader population. In addition, a few participants (2/10, 20%) felt that some photographs appeared too professional and posed, making it difficult to identify with them.

##### Feedback on the Workbook

Participants positively evaluated the inclusion of a Workbook, highlighting the value of receiving feedback from a psychologist, which conveyed a sense of personalized conversation. They found the idea of having a professional behind the technology to be innovative and supportive, which could increase the use of it as a meaningful support for those struggling with postpartum depression:

For me personally, the fact that one has the option of writing and receiving feedback already gives me the feeling that I am speaking to someone. It’s not like a predetermined survey, and then they give you an automated result, so I like the idea of having someone who is actually going to read and respond something personalized somehow.

##### Feedback on the Short Version of the Prototype

All participants (10/10, 100%) found the short version of the prototype user-friendly and intuitive. The colors and design were well received and perceived as “pleasing to the eye.” The font size and amount of text were considered appropriate and easy to read. The quality, depth, and comprehensiveness of the information provided and the feasibility of reading 1 module per week were well evaluated:

It is super friendly, the text does not seem so much to me, and it is easy to read; It is not saturated with things; sometimes they [apps] are saturated with images and icons; this one is simple, so it makes me want to read it.

You can tell it [the information] was written by someone who wanted it to be easy for you to read and understand.

The inclusion of fictional characters and the presence of features like the favorites or saved button, the infographic (guide for healing), and the reading for loved ones received positive feedback:

I found it super positive that there are cases about depression or symptoms, such as stories, because that also helps you to identify yourself with some stories and thus also say, “heck, I’m not the only one that happens to me,” or “maybe I have these symptoms.”

[I]t is important [the reading for loved ones] because often relatives, instead of helping, make it worse, make you feel bad or sometimes say super unfortunate things.

Some participants expressed a desire for specific information or features that were not included in the prototype they explored. These desires involved accessing references for the information provided, including a resources section containing articles or books, and integrating a depression and anxiety symptoms questionnaire with automated feedback.

##### Feedback on the Topics of the Infographics

Participants found most of the mentioned topics (1-6) relevant for an infographic. In each group, on average, every subject was rated above 4.3 (on a 1-5 scale), except for “Coping with postpartum rage,” which had an average rating of 3.3 in 1 focus group and 5 in the other. A few participants (2/10, 20%) suggested adding infographics on returning to work after maternity leave and the baby’s sleep.

#### Requirements Sessions

In response to the feedback received, we adjusted the illustrations, ensuring that they depict a broader range of emotions and incorporate other members of the mother’s social network. We also introduced an infographic addressing returning to work after maternity leave. Furthermore, we have included a section that contains references for all the information used and another one that provides a list of websites and hotlines offering support for maternity and mental health, including websites with evidence-based information about baby’s sleep.

#### User-Based Usability Evaluation

##### Feedback on Design, Sections, and Features

The color palette and the inclusion of illustrations and photographs accompanying the text were positively evaluated:

I find the presentation of colors kind of tender, calm... that pastel purple is a calm color; it is not an aggressive color because there are applications that have aggressive colors, but not this one.

It is nice to have photographs between the text, so it’s not so overloaded with text.

All participants (5/5, 100%) liked the illustrations and photographs, finding them “pretty” and “attractive.” They also highly appreciated the fictional mothers narrating their stories and exemplifying the content.

The length of the text was deemed appropriate. All participants (5/5, 100%) found the text size suitable (none had vision problems). All the sections of the prototype (Modules, Favorites, Workbook, Infographics, About, Resources, and Reading for loved ones) were evaluated as valuable.

##### Feedback on Prototype’s User-Friendliness

All participants (5/5, 100%) mentioned that they found the prototype user-friendly. When asked to rate the prototype’s user-friendliness from 1 (not friendly) to 10 (very friendly), participants gave ratings between 7 and 10 (mean 8.6, SD 1.1). Some suggestions arose when asked what was needed to rate it the maximum score: including a night mode, increasing the text font size to be more inclusive to visually impaired people, enabling a quick return to where they left off in the module when returning to the platform, including a depression or anxiety symptoms questionnaire, and displaying the logos of the institutions listed in the resources section to make them easier to identify. Each suggestion was mentioned by 1 or 2 participants:

It is friendly, easy to log in, easy to follow the steps.

It seems very friendly to me; the only thing I would need would be the night mode because I will probably read it at night... 9, as I told you, the only thing lacking for me is that.

##### Willingness to Use the Platform if Struggling With Depression Symptoms

All participants (5/5, 100%) said they would use the platform if struggling with low mood or depression symptoms. They valued its user-friendliness; its friendliness in tone or images; the content provided; having all the information on perinatal mental health and management in one place; having the option to interact with an e-coach; and trusting the platform because it was designed by a Chilean team of professionals, perceived as trustworthy:

Yes, because it looks like it has all the practical information on how to deal with those symptoms and in a platform made by a team that makes you trust them... instead of Googling it.

##### Willingness to Recommend the Platform to a Friend

All participants (5/5, 100%) expressed strong receptivity toward recommending the prototype to a friend struggling with postpartum low mood or depression symptoms, using words like “definitely,” “totally,” and “sure*.*” The information provided was highlighted as its primary value.

Yes, so she can inform herself and read about her psychological process, understand herself more, and at what point she should seek help or how to communicate it to her family.

#### Requirements Sessions

The results of the interviews were discussed with the project development team. Given the positive feedback on the design and user-friendliness of the Figma prototype, it was decided to develop the actual version of the IBI using the Figma prototype as the blueprint. The intervention had to be accessible by smartphone, enable communication with the e-coach, and not necessarily require installation. It was decided to create a web application, which is a software application that runs on a web browser and is accessed over the internet. Unlike traditional applications that must be installed on a computer or mobile device, web applications do not require installation. Users can access them directly through a web browser. Web applications are designed to provide interactive functionality and offer various features and services, similar to mobile apps. For the TP, we requested the participants to add a shortcut to the web app on their phone’s screen, enabling them to access it quickly, such as a mobile app.

The points for improvement were discussed. While the project team agreed that adding a night mode would be beneficial, it was decided not to include it in the current version due to time and resource constraints, though it is planned for future updates. The logos of the websites and hotlines from the resources section were included next to their names.

#### TP Phase

##### Detection of Bugs and Usability Improvements

All 10 participants registered on the web app. A total of 4 (40%) participants completed all main modules, and 2 (20%) completed the additional modules. While 7 (70%) participants submitted at least 1 exercise, 2 (20%) submitted the exercises of all the main modules, and 1 (10%) also sent the exercises of the extra modules. The therapist provided feedback on the exercises within 48 hours. Throughout the study, participants reported 8 bugs, all related to nonfunctional clicks. The software developers promptly addressed and resolved each issue.

In addition, 2 suggestions were made to improve the web app’s usability concerning the exercises. At that moment, users could only know they received the e-coach’s feedback on their exercises by navigating to the Workbook and finding the specific exercise they submitted, where the feedback is displayed beneath their text. In response, 2 participants expressed their desire for a notification system to promptly inform them of feedback pending reading upon accessing the web app. The other suggestion came from 2 participants, who proposed enabling an autosave function when doing the exercises. They mentioned encountering instances when they could not finish and submit an exercise, which meant redoing the entire exercise from scratch later. The first suggestion has been addressed with a visual notification system within the web app, while the second suggestion will be considered for a future update.

##### Satisfaction and Engagement With the Web App

The overall CSQ-8 sum score averaged 28.14 (SD 4.29), with only 1 participant assigning the lowest possible score to a single item. The items, ranked from the highest to the lowest mean (in a range from 1 to 4), are as follows: overall satisfaction (3.71), quality of service (3.71), deal with problems (3.71), amount of help (3.71), recommend to a friend (3.57), come back (3.57), type of service (3.28), and met needs (2.85) (see Table S2 in Multimedia Appendix 3 for CSQ-8 item response frequency).

The mean total TWEETS score was 3.20 (SD 0.55), and no participant assigned the lowest possible score to any of the items. The items, ranked from the highest to the lowest mean (in a range from 0 to 4), are as follows: “I enjoyed using MTE” (3.57), “MTE takes me little effort to use” (3.57), “I was able to use MTE as often as needed to improve my mood” (3.42), “I enjoyed seeing the progress I made in MTE” (3.36), “MTE helped me to get more insight into my thoughts and emotions” (3.28), “MTE fits me as a person” (3.23), “MTE made it easier for me to work on improving my mood” (3.28), “MTE motivated me to improve my mood” (3.14), and “MTE was part of my daily routine” (2.00; see Table S3 in Multimedia Appendix 3 for TWEETS item response frequency).

### The Internet-Based Intervention

**Figure 1:**
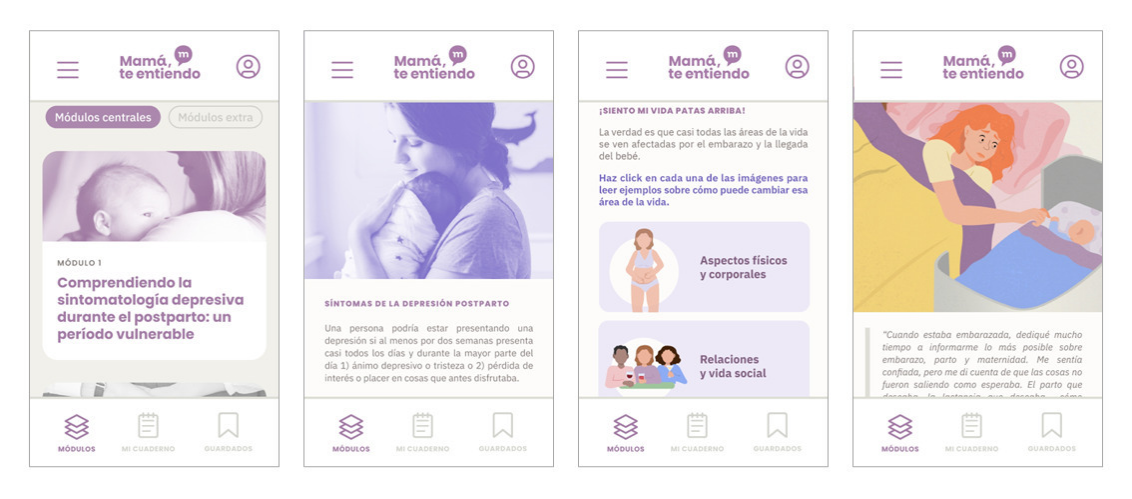
A sample of screenshots of the Mamá, te entiendo web app.

In this subsection, we describe the resulting IBI. Mamá, te entiendo (“Mom, I get you”) is a CBT web app intervention that includes elements from attachment and mentalization theories. The intervention consists of 6 main sequential modules and 3 extra modules (Table 4). Five case examples of mothers illustrate depressive symptoms and techniques. There is also an introductory module, and a guide about maintaining changes (relapse prevention) is displayed when the last main module is completed. Women can select any part of the content as “add to favorites” and save it in a “Favorites” section. The optional homework exercises are included in the modules alongside the related content and in a separate “Workbook” section. Participants receive feedback from an e-coach through the web app. They can also contact the e-coach to resolve doubts about the content and exercises. A sample of screenshots of the web app is presented in Figure 1.

Another section features infographics covering various pertinent postpartum mental health topics, including (1) a guide to healing; (2) strengthening your support network; (3) coping with postpartum rage; (4) navigating changes in your postpartum body; (5) handling doubts, comparisons, and information overload (empowering yourself as a mother); (6) mentalizing your baby; (7) nurturing the couple’s relationship during the postpartum period; and (8) returning to work after maternity leave. In addition, the app includes a shareable document with insights on supporting a loved one struggling with symptoms of depression. A resources section provides links to websites and hotlines for mental health and maternity support (most of them from institutions within the country). The app also dedicates a section to introduce the team responsible for its development and references for its content. Finally, a contact section enables participants to send messages to the IT support team in case of technical issues.

**Table 4:** Mamá, te entiendo modules.

Module			Topic		Exercise techniques
0			Introduction to Mamá, te entiendo functioning (no therapeutic content)		—^a^
1			Psychoeducation on depression		—
2			How the CBT^b^ approach works		Goal setting and tracking dysfunctional thoughts
3			Identifying thinking errors		Tracking dysfunctional thoughts and identifying thinking errors
4			Cognitive restructuring		“Best friend” technique, examining the evidence, and the possibility pie
5			Problem-solving		Problem-solving strategy
6			Behavioral activation		Activity scheduling
**Relapse prevention guide**					
	Extra 1	Psychoeducation on anxiety		—	
	Extra 2	Exposure strategies for addressing anxiety		Exposure strategies	
	Extra 3	Communicational skills		Assertive communication plan	

^a^Not applicable.

^b^CBT: cognitive behavioral therapy.

## Discussion

### Principal Findings

This study aimed to create a user-friendly technology for perinatal depression symptoms that aligns with stakeholders’ values and to evaluate the development process. The intervention development followed a user-centered design approach, actively involving perinatal women and health care professionals in providing input and feedback. The process was iterative and used a variety of research methods. The invaluable insights from stakeholders, combined with a literature study, shaped the web app’s content, layout, and features.

The overall development process was satisfactory. The development team gained valuable insights into the importance of iterative testing and user input. Different research activities were used to achieve a wide range of goals, providing crucial insights into users and the context, which guided the refinements of the web app. Each step was built upon the knowledge gained from earlier stages and informed the next steps. The literature study helped us understand the potential of addressing perinatal depression through a psychological IBI in Chile; solved questions related to therapeutic content; and offered design recommendations, such as including fictional characters. Surveys provided insight into attitudes toward existing perinatal health apps and identified the needs and preferences for an “ideal” technology for the perinatal period. The first round of focus groups offered insights into stakeholders’ attitudes toward a potential intervention for reducing perinatal depression symptoms and enhancing well-being as well as identifying values and requirements for such an intervention. The second round of focus groups helped collect input to improve the prototype. UIs provided valuable feedback on the web app’s user-friendliness and users’ initial impressions. The TP assessed the app’s functionality and provided initial feedback on women’s satisfaction and engagement.

However, it is essential to recognize that a one-size-fits-all approach does not apply to development processes. Our method, which included several standard methods, could have been further enhanced by integrating innovative and participatory techniques, as Kip et al [5] recommend. By contrast, we faced challenges in harmonizing contributions from multiple participants, selecting relevant information and materials, and working within time and resource constraints. These challenges were addressed through proactive problem-solving and collaborative efforts among team members. For example, while there was a high demand for a feature allowing interactions with other mothers, its implementation was not feasible not only due to logistical constraints and limited resources but it diverged from our objectives. The multidisciplinary research team and requirement sessions were crucial in addressing these emerging challenges. However, not all team members could always be present at meetings due to time, language, and time zone constraints. In some cases, debriefing sessions were held with specific team members to discuss project progress and decisions, which were then addressed in subsequent meetings with other team members. Nevertheless, it would not have been practical to have all the meetings with all team members, as some were highly technical or involved decisions that did not require input from the entire team. However, the project’s principal investigator attended all meetings.

We also learned important lessons regarding the evaluation of materials. For instance, negative feedback regarding the photographs mainly emerged when they were presented in isolation during focus groups, but no such comments arose when viewing them within the prototype context. The inclusion of these photographs was well received, likely because they resembled those used in other perinatal mobile apps or websites, providing a different perspective when viewed in context. The last underscores the importance of not relying solely on out-of-context feedback and the need to incorporate multiple methods while verifying earlier findings.

Postpartum women appreciated the integration of realistic illustrations and photographs, which enhanced their connection with the material. Furthermore, including 5 fictional characters throughout the content effectively conveyed the requirements of showing the “real motherhood” and incorporating testimonials from women. This approach aligns with other IBIs for perinatal depression that have shown potential efficacy [32,33,38]. Such inclusion helps women dispel “motherhood myths” and normalize and validate their thoughts and experiences with motherhood, as highlighted in qualitative studies related to IBIs [29,49].

In addition, the sense of “being on the same page” may have reduced this target group’s need for personalization and tailoring. Aside from the personalized feedback provided by the e-coach, the optional nature of the exercises, and the availability of extra optional modules, participants did not emphasize the importance of personalization in our study. Instead, “group-based” content personalization, designed to target the intervention as a whole for postpartum women with depression symptoms, appeared to be more relevant. The last suggests that a general intervention for depression (without explicitly addressing postpartum issues) may not appeal to this target group. The absence of personalization requests may also be due to the novelty of the intervention, as there are no similar web-based interventions for adults in the country, leaving women uncertain about what to expect regarding personalization. Nevertheless, we believe that exploring additional personalization strategies in the future, as suggested by Hornstein et al [50], is a worthwhile endeavor. Adapting the content and design of an intervention to users’ needs aims to maximize adherence, engagement, and, ultimately, treatment efficacy, as emphasized by Ludden et al [51] and Kelders [52].

Future plans also include the consideration of implementing a night mode for reading in low-light conditions and adding an autosave function to prevent users from losing progress. In addition, we will explore the possibility of conducting a photo shoot with “Chilean moms” to enhance the representation of images in the intervention. Despite these considerations, the positive feedback on content and features, along with favorable assessments of user-friendliness, satisfaction, and engagement, supports the web app’s readiness for further testing. Notably, a feasibility and acceptability study has already been conducted, yielding promising results that have recently been published [6]. Motivated by these findings and participants’ acceptability feedback, we are updating the intervention and proceeding with a larger-scale study to continue evaluating its impact.

### Study Limitations

Our study has some limitations concerning the samples of perinatal women. One limitation is that the presence of depressive symptoms was not used as an inclusion criterion for participants in the various research activities. While at least 1 participant in each focus group, interview, and TP mentioned undergoing treatment for depression, actively including a more significant number of women with depressive symptoms might have yielded more focused input from this group. Nevertheless, given that the recruitment material emphasized an intervention to enhance perinatal mental health, we believed that it would naturally attract a significant proportion of individuals grappling with this issue. Another limitation is that the target users who participated in the contextual inquiry (and likely in the value specification and design phases) were primarily highly educated women. We did not assess the education level during the value specification and design phases, but since the recruitment process resembled that of the contextual inquiry, the samples may have similarities. Another limitation concerning the study participants is that all procedures were performed with small groups of participants, potentially limiting generalizability. Nevertheless, it is important to note that our findings should be considered a preliminary step and must be confirmed within the specific audience for the intervention. This iterative approach aligns with the fundamental principles outlined in the CeHRes Roadmap [4].

Another limitation is the limited involvement of health care providers in the development process. This decision was made during the contextual inquiry, where we focused on research as the implementation setting. However, if we choose to expand the development of this intervention for a different implementation context, it is imperative to include health care providers in the process. In addition, fathers or lesbian partners did not contribute to the development process. Partners were invited to participate in 2 focus groups during the value specification phase, but the women attended these sessions individually. The last occurred despite our efforts to involve partners, highlighting a gap in our recruitment strategy. We did not initially plan to organize a focus group exclusively for partners. We acknowledge that this may limit the scope of our findings and potentially introduce bias, as partners play a significant role in the perinatal experience. Although participants appreciated the fact that the web app was designed “for them,” considering the importance of the couple’s relationship during the transition to parenthood and the expressed desires of mothers to involve their partners in some way, it may be beneficial to include partners as stakeholders in the future. Future formative research should involve partners as active stakeholders to enrich the intervention’s content and appeal and to ensure that it supports the dynamics of the couple’s relationship during the transition to parenthood.

Finally, in this study, we did not evaluate the impact or effectiveness of the intervention, so we cannot determine if it successfully reduces depressive symptoms in the target group. Future research is necessary to assess if users will use the intervention and if it achieves the intended effects. To improve the intervention’s design and effectiveness, it is crucial to use those methods that objectively measure use, gather qualitative feedback on user satisfaction and acceptability, and assess clinical effectiveness [53].

### Conclusions

In conclusion, the IBI Mamá, te entiendo is a significant step toward addressing the pressing issue of perinatal depression in Chile. Through a comprehensive and user-centered design process, we aimed to create an accessible and culturally sensitive platform to support postpartum women experiencing depression symptoms. The integration of CBT principles and elements from attachment and mentalization theories and the inclusion of realistic case examples and infographics have resulted in a well-rounded intervention that targets the unique challenges that mothers face during the postpartum period. The web app offers evidence-based strategies and psychoeducation in a user-friendly format, potentially empowering women to manage depression and anxiety symptoms. An e-coach feature is included to bridge the gap between professional care and self-help, offering additional support. Positive feedback from perinatal women during the intervention development indicates its potential as a valuable mental health tool. If effective, it could significantly improve perinatal mental health in Chile and serve as a model for similar interventions in other Latin American countries.

## Appendix

Multimedia Appendix 1 Focus groups and usability interview scripts.

Multimedia Appendix 2 Material presented in focus groups 9 and 10.

Multimedia Appendix 3 Supplementary tables.

## Abbreviations

CBT: cognitive behavioral therapy
CeHRes: Center for eHealth Research and Disease Management
CSQ-8: Client Satisfaction questionnaire-8
EPDS: Edinburgh Postnatal Depression Scale
IBI: internet-based intervention
TP: technical pilot
TWEETS: Twente Engagement With eHealth Technologies Scale
UI: usability interview

## Data Availability

The data sets generated during and analyzed during this study are available from the corresponding author on reasonable request.

## References

[1] Feldman N, Perret S (2023). Digital mental health for postpartum women: perils, pitfalls, and promise. NPJ Digit Med.

[2] Lau Y, Htun TP, Wong SN, Tam WS, Klainin-Yobas P (2017). Therapist-supported internet-based cognitive behavior therapy for stress, anxiety, and depressive symptoms among postpartum women: a systematic review and meta-analysis. J Med Internet Res.

[3] Moshe I, Terhorst Y, Philippi P, Domhardt M, Cuijpers P, Cristea I, Pulkki-Råback L, Baumeister H, Sander LB (2021). Digital interventions for the treatment of depression: a meta-analytic review. Psychol Bull.

[4] van Gemert-Pijnen L, Kelders SM, Kip H, Sanderman R (2018). eHealth Research, Theory and Development: A Multi-Disciplinary Approach.

[5] Kip H, Keizer J, da Silva MC, Beerlage-de Jong N, Köhle N, Kelders SM (2022). Methods for human-centered ehealth development: narrative scoping review. J Med Internet Res.

[6] Franco P, Olhaberry M, Kelders S, Muzard A, Cuijpers P (2024). Guided web app intervention for reducing symptoms of depression in postpartum women: results of a feasibility randomized controlled trial. Internet Interv.

[7] Franco P, Olhaberry M, Kelders S, Muzard A (2023). A Chilean survey of perinatal women and health care professionals' views towards perinatal apps. Mhealth.

[8] Drisko J, Maschi T (2015). Content Analysis.

[9] Think bigger. Build faster. Figma.

[10] Arsand E, Demiris G (2008). User-centered methods for designing patient-centric self-help tools. Inform Health Soc Care.

[11] Jadresic E, Araya R, Jara C (1995). Validation of the Edinburgh Postnatal Depression Scale (EPDS) in Chilean postpartum women. J Psychosom Obstet Gynaecol.

[12] Vázquez FL, Torres Á, Otero P, Blanco V, Clifford Attkisson C (2017). Psychometric properties of the Castilian Spanish version of the client satisfaction questionnaire (CSQ-8). Curr Psychol.

[13] Kelders SM, Kip H, Greeff J (2020). Psychometric evaluation of the TWente Engagement with Ehealth Technologies Scale (TWEETS): evaluation study. J Med Internet Res.

[14] (2014). Protocolo de detección de la depresión durante el embarazo y posparto y apoyo al tratamiento. Ministerio de Salud.

[15] (2017). Guías Clínicas AUGE: para el tratamiento de la depresión en personas mayores de 15 años: actualización en psicoterapia. Ministerio de Salud.

[16] Rojas G, Guajardo V, Martínez P, Fritsch R (2018). [Screening and barriers for treatment of postpartum depression in Chilean public primary health care centers]. Rev Med Chil.

[17] Rojas G, Santelices MP, Martínez P, Tomicic A, Reinel M, Olhaberry M, Krause M (2015). [Barriers restricting postpartum depression treatment in Chile]. Rev Med Chil.

[18] De la Parra G, Errázuriz P, Gómez-Barris E, Zúñiga AK (2019). Propuesta para una psicoterapia efectiva en atención primaria: un modelo basado en la experiencia y la evidencia empírica. Temas de la Agenda Pública.

[19] Cuijpers P, Smit F (2008). [Subclinical depression: a clinically relevant condition?]. Tijdschr Psychiatr.

[20] Volz HP, Stirnweiß J, Kasper S, Möller HJ, Seifritz E (2023). Subthreshold depression - concept, operationalisation and epidemiological data. A scoping review. Int J Psychiatry Clin Pract.

[21] Hao X, Jia Y, Chen J, Zou C, Jiang C (2023). Subthreshold depression: a systematic review and network meta-analysis of non-pharmacological interventions. Neuropsychiatr Dis Treat.

[22] Coo Calcagni S, Mira Olivos A, García Valdés MI, Zamudio Berrocal P (2021). [Perinatal mental health in chilean mothers]. Andes Pediatr.

[23] Ramakrishna S, Cooklin AR, Leach LS (2019). Comorbid anxiety and depression: a community-based study examining symptomology and correlates during the postpartum period. J Reprod Infant Psychol.

[24] Franco P, Olhaberry M, Muzard A, Lara MA, Cuijpers P, Martínez V, Miranda-Castillo C (2022). The potential of internet-based psychological interventions for perinatal depression prevention and treatment. Prevention and Early Treatment of Depression Through the Life Course.

[25] O'Mahen HA, Woodford J, McGinley J, Warren FC, Richards DA, Lynch TR, Taylor RS (2013). Internet-based behavioral activation--treatment for postnatal depression (Netmums): a randomized controlled trial. J Affect Disord.

[26] O'Mahen HA, Richards DA, Woodford J, Wilkinson E, McGinley J, Taylor RS, Warren FC (2014). Netmums: a phase II randomized controlled trial of a guided internet behavioural activation treatment for postpartum depression. Psychol Med.

[27] Barrera AZ, Wickham RE, Muñoz RF (2015). Online prevention of postpartum depression for Spanish- and English-speaking pregnant women: a pilot randomized controlled trial. Internet Interv.

[28] Milgrom J, Danaher BG, Gemmill AW, Holt C, Holt CJ, Seeley JR, Tyler MS, Ross J, Ericksen J (2016). Internet cognitive behavioral therapy for women with postnatal depression: a randomized controlled trial of MumMoodBooster. J Med Internet Res.

[29] Pugh NE, Hadjistavropoulos HD, Dirkse D (2016). A randomised controlled trial of therapist-assisted, internet-delivered cognitive behavior therapy for women with maternal depression. PLoS One.

[30] Forsell E, Bendix M, Holländare F, Szymanska von Schultz B, Nasiell J, Blomdahl-Wetterholm M, Eriksson C, Kvarned S, Lindau van der Linden J, Söderberg E, Jokinen J, Wide K, Kaldo V (2017). Internet delivered cognitive behavior therapy for antenatal depression: a randomised controlled trial. J Affect Disord.

[31] Haga SM, Drozd F, Lisøy C, Wentzel-Larsen T, Slinning K (2019). Mamma Mia - a randomized controlled trial of an internet-based intervention for perinatal depression. Psychol Med.

[32] Loughnan SA, Butler C, Sie AA, Grierson AB, Chen AZ, Hobbs MJ, Joubert AE, Haskelberg H, Mahoney A, Holt C, Gemmill AW, Milgrom J, Austin MP, Andrews G, Newby JM (2019). A randomised controlled trial of 'MUMentum postnatal': internet-delivered cognitive behavioural therapy for anxiety and depression in postpartum women. Behav Res Ther.

[33] Loughnan SA, Sie A, Hobbs MJ, Joubert AE, Smith J, Haskelberg H, Mahoney AE, Kladnitski N, Holt CJ, Milgrom J, Austin MP, Andrews G, Newby JM (2019). A randomized controlled trial of 'MUMentum Pregnancy': internet-delivered cognitive behavioral therapy program for antenatal anxiety and depression. J Affect Disord.

[34] Sawyer A, Kaim A, Le HN, McDonald D, Mittinty M, Lynch J, Sawyer M (2019). The effectiveness of an app-based nurse-moderated program for new mothers with depression and parenting problems (eMums Plus): pragmatic randomized controlled trial. J Med Internet Res.

[35] Yang M, Jia G, Sun S, Ye C, Zhang R, Yu X (2019). Effects of an online mindfulness intervention focusing on attention monitoring and acceptance in pregnant women: a randomized controlled trial. J Midwifery Womens Health.

[36] Heller HM, Hoogendoorn AW, Honig A, Broekman BF, van Straten A (2020). The effectiveness of a guided internet-based tool for the treatment of depression and anxiety in pregnancy (MamaKits Online): randomized controlled trial. J Med Internet Res.

[37] Fonseca A, Alves S, Monteiro F, Gorayeb R, Canavarro MC (2020). Be a mom, a web-based intervention to prevent postpartum depression: results from a pilot randomized controlled trial. Behav Ther.

[38] Jannati N, Mazhari S, Ahmadian L, Mirzaee M (2020). Effectiveness of an app-based cognitive behavioral therapy program for postpartum depression in primary care: a randomized controlled trial. Int J Med Inform.

[39] Sun Y, Li Y, Wang J, Chen Q, Bazzano AN, Cao F (2021). Effectiveness of smartphone-based mindfulness training on maternal perinatal depression: randomized controlled trial. J Med Internet Res.

[40] Baumeister H, Reichler L, Munzinger M, Lin J (2014). The impact of guidance on internet-based mental health interventions — a systematic review. Internet Interv.

[41] Karyotaki E, Efthimiou O, Miguel C, Bermpohl FM, Furukawa TA, Cuijpers P, Riper H, Patel V, Mira A, Gemmil AW, Yeung AS, Lange A, Williams AD, Mackinnon A, Geraedts A, van Straten A, Meyer B, Björkelund C, Knaevelsrud C, Beevers CG, Botella C, Strunk DR, Mohr DC, Ebert DD, Kessler D, Richards D, Littlewood E, Forsell E, Feng F, Wang F, Andersson G, Hadjistavropoulos H, Christensen H, Ezawa ID, Choi I, Rosso IM, Klein JP, Shumake J, Garcia-Campayo J, Milgrom J, Smith J, Montero-Marin J, Newby JM, Bretón-López J, Schneider J, Vernmark K, Bücker L, Sheeber LB, Warmerdam L, Farrer L, Heinrich M, Huibers MJ, Kivi M, Kraepelien M, Forand NR, Pugh N, Lindefors N, Lintvedt O, Zagorscak P, Carlbring P, Phillips R, Johansson R, Kessler RC, Brabyn S, Perini S, Rauch SL, Gilbody S, Moritz S, Berger T, Pop V, Kaldo V, Spek V, Forsell Y, Individual Patient Data Meta-Analyses for Depression (IPDMA-DE) Collaboration (2021). Internet-based cognitive behavioral therapy for depression: a systematic review and individual patient data network meta-analysis. JAMA Psychiatry.

[42] Spanhel K, Balci S, Feldhahn F, Bengel J, Baumeister H, Sander LB (2021). Cultural adaptation of internet- and mobile-based interventions for mental disorders: a systematic review. NPJ Digit Med.

[43] Oinas-Kukkonen H, Harjumaa M (2009). Persuasive systems design: key issues, process model, and system features. Commun Assoc Inf Syst.

[44] Wildeboer G, Kelders SM, van Gemert-Pijnen JE (2016). The relationship between persuasive technology principles, adherence and effect of web-based interventions for mental health: a meta-analysis. Int J Med Inform.

[45] Sleed M, Li ET, Vainieri I, Midgley N (2023). The evidence-base for psychodynamic interventions with children under 5 years of age and their caregivers: a systematic review and meta-analysis. J Infant Child Adolesc Psychother.

[46] Rosenblum K, Lawler J, Alfafara E, Miller N, Schuster M, Muzik M (2018). Improving maternal representations in high-risk mothers: a randomized, controlled trial of the mom power parenting intervention. Child Psychiatry Hum Dev.

[47] Menashe-Grinberg A, Shneor S, Meiri G, Atzaba-Poria N (2022). Improving the parent-child relationship and child adjustment through parental reflective functioning group intervention. Attach Hum Dev.

[48] Green SM, Frey BN, Donegan E, McCabe RE (2018). Cognitive Behavioral Therapy for Anxiety and Depression During Pregnancy and Beyond: How to Manage Symptoms and Maximize Well-Being.

[49] O'Mahen HA, Grieve H, Jones J, McGinley J, Woodford J, Wilkinson EL (2015). Women's experiences of factors affecting treatment engagement and adherence in internet delivered Behavioural Activation for Postnatal Depression. Internet Interv.

[50] Hornstein S, Zantvoort K, Lueken U, Funk B, Hilbert K (2023). Personalization strategies in digital mental health interventions: a systematic review and conceptual framework for depressive symptoms. Front Digit Health.

[51] Ludden GD, van Rompay TJ, Kelders SM, van Gemert-Pijnen JE (2015). How to increase reach and adherence of web-based interventions: a design research viewpoint. J Med Internet Res.

[52] Kelders SM, Van Zyl L, Rothmann S Sr (2019). Design for engagement of online positive psychology interventions. Positive Psychological Intervention Design and Protocols for Multi-Cultural Contexts.

[53] Kelders SM, Pots WT, Oskam MJ, Bohlmeijer ET, van Gemert-Pijnen JE (2013). Development of a web-based intervention for the indicated prevention of depression. BMC Med Inform Decis Mak.

