# Supplementary material for: Developing a Guided Web App for Postpartum Depression Symptoms: User-Centered Design Approach
Source: JMIR Form Res. 2024 Aug 19;8:e56319. doi: 10.2196/56319 (PMC11369531; doi:10.2196/56319)
Supplement: Multimedia Appendix 1 [file formative_v8i1e56319_app1.docx]

**First-Round of Focus Groups' Script (Focus Groups 1 to 8)**

Instructions:

In this focus group, we are interested in hearing your ideas, perceptions, and visions about what this intervention could look like. There are no expected responses; your views are important, and whatever comes to mind first in response to the questions is important.

Explanation about internet-based interventions studied.

Questions about impressions:

a. What is your impression of such an intervention?

b. Would you use such an intervention? / Would you recommend such an intervention?

Questions about technology:

a. What features would it have? (e.g., photos, videos, text...)

b. Should this technology provide new information periodically? If so, how often? How long should it take to read the information?

c. Should this technology suggest or propose activities to do? If so, what should these activities be like? How often?

d. Should this technology allow contact with a therapist? If so, how should that contact be? When?

e. Should it allow contact with other mothers?

f. Should it include the partner? In what way?

g. Should this technology allow data to be saved? (e.g., from questionnaires that one answers)

h. What features should this technology have to engage women, that is, make them want to and enjoy accessing/using it? / What features should it NOT have?

i. What should this technology look like visually? (design, layout)

j. If you had to choose between having it during pregnancy or postpartum, which would you choose?

In the focus group with women who were treated for postpartum depression: further inquire about what features the intervention and the intervention coaching should have based on their therapeutic experiences.

In the focus group with perinatal health professionals: inquire about the most common reasons for consultation among women in the perinatal period.

**Second-round of Focus Groups' Script (Focus Groups 9 & 10)**

Instructions:

In this focus group, we are interested in hearing your opinions on proposals we have developed based on the information gathered in previous focus groups. There are no expected responses; your views are important, and whatever comes to mind first in response to the proposals we will present is important.

- Evaluate impressions regarding the illustrations (aesthetics, representativeness, emotional tone, etc.).
- Evaluate impressions regarding the photographs (aesthetics, representativeness, emotional tone, etc.).
- Evaluate impressions of the workbook: What do you think about having reflection exercises of this style to practice the techniques? What do you think about receiving feedback from a psychologist? There will also be a section to ask questions about the app's content and the exercises. What do you think about this?
- Evaluate impressions of the prototype on their smartphones:

a. First impressions?

b. Text and length: What do you think about the font size? What do you think about the amount of text in the sections? What do you think about the amount of text in one module? Is it feasible to review that amount of text and complete a reflection exercise in one week?

c. Favorites section: What do you think about having a favorites section? How important is it to include this section, on a scale of 1 to 5?

d. Infographics: What do you think about addressing each of these topics from 1 (not relevant) to 5 (very relevant)? Would you add any (related to mental health)?

e. Reading for loved ones: What do you think about having a reading for loved ones on how to support a mom's mental health postpartum? Would you share it with people in your environment? What would you like it to say?

f. Resources section: What would you like to find in the resources section?

g. Creator team: Do you want to know who is behind the app? What would you like to know about these people?

h. Therapist: What would you like to know about the psychotherapists who will respond to the messages?

**Usability Interview Script**

- Interviewer Introduction
- Interviewee Introduction

a. Age

b. Baby number

c. Baby's age

- Introduction to the Project
- Introduction about the intervention
- Usability Testing:

a. Link to the clickable prototype.

b. Mention that not all content can be reviewed; focus on prototype exploration rather than content review.

c. Explain the "think aloud" method and its duration (15 minutes).

d. Ensure the following components have been evaluated:

- Illustrations
- Photographs
- Exercise Workbook
- Text (length and size)
- Favorites section
- Infographics
- Reading for loved ones
- Resources section
- Team section

e. Final Questions:

- On a scale of 1 to 10, how user-friendly do you find the prototype? Why?
- If you were feeling down, do you think you would use this technology? Why?
- Would you recommend this technology to a friend who is feeling down during her postpartum period? Why?
