# Supplementary material for: Developing a Guided Web App for Postpartum Depression Symptoms: User-Centered Design Approach
Source: JMIR Form Res. 2024 Aug 19;8:e56319. doi: 10.2196/56319 (PMC11369531; doi:10.2196/56319)

**Multimedia Appendix 2**

**Material presented in Focus Groups 9 and 10**

1. Set of illustrations:


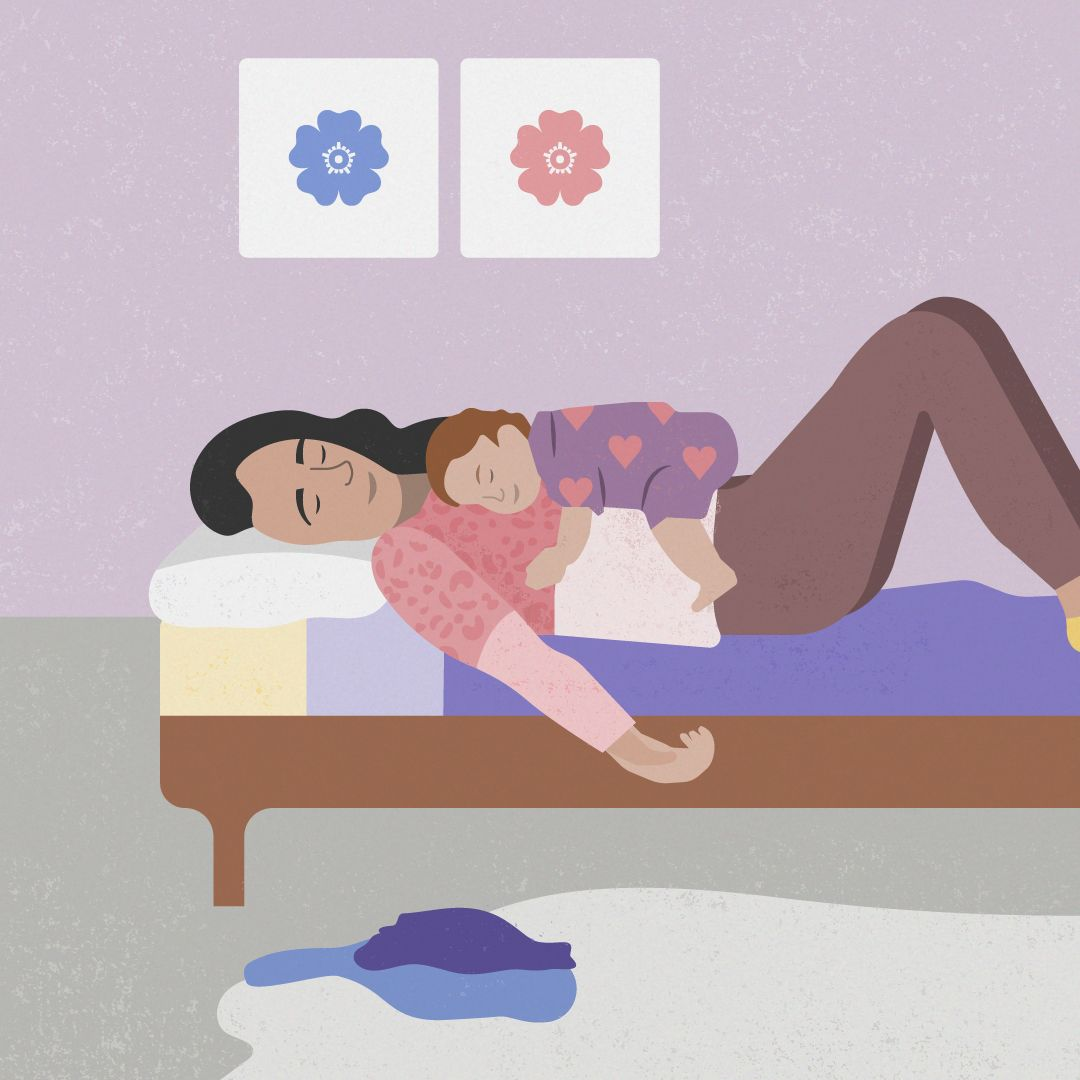

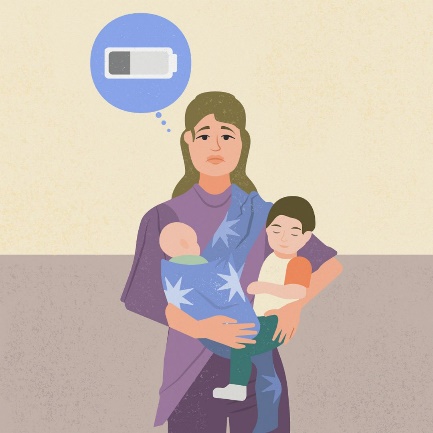

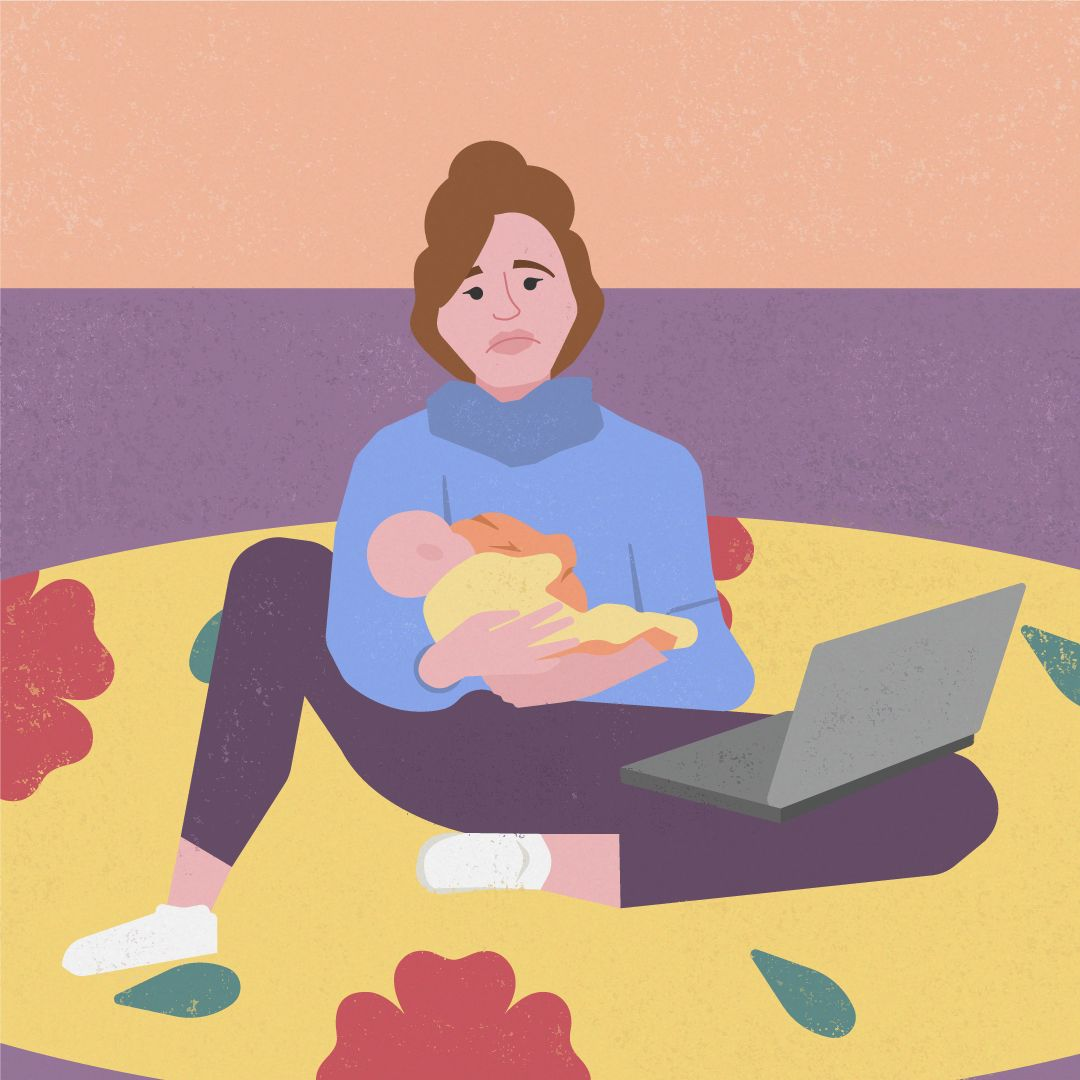

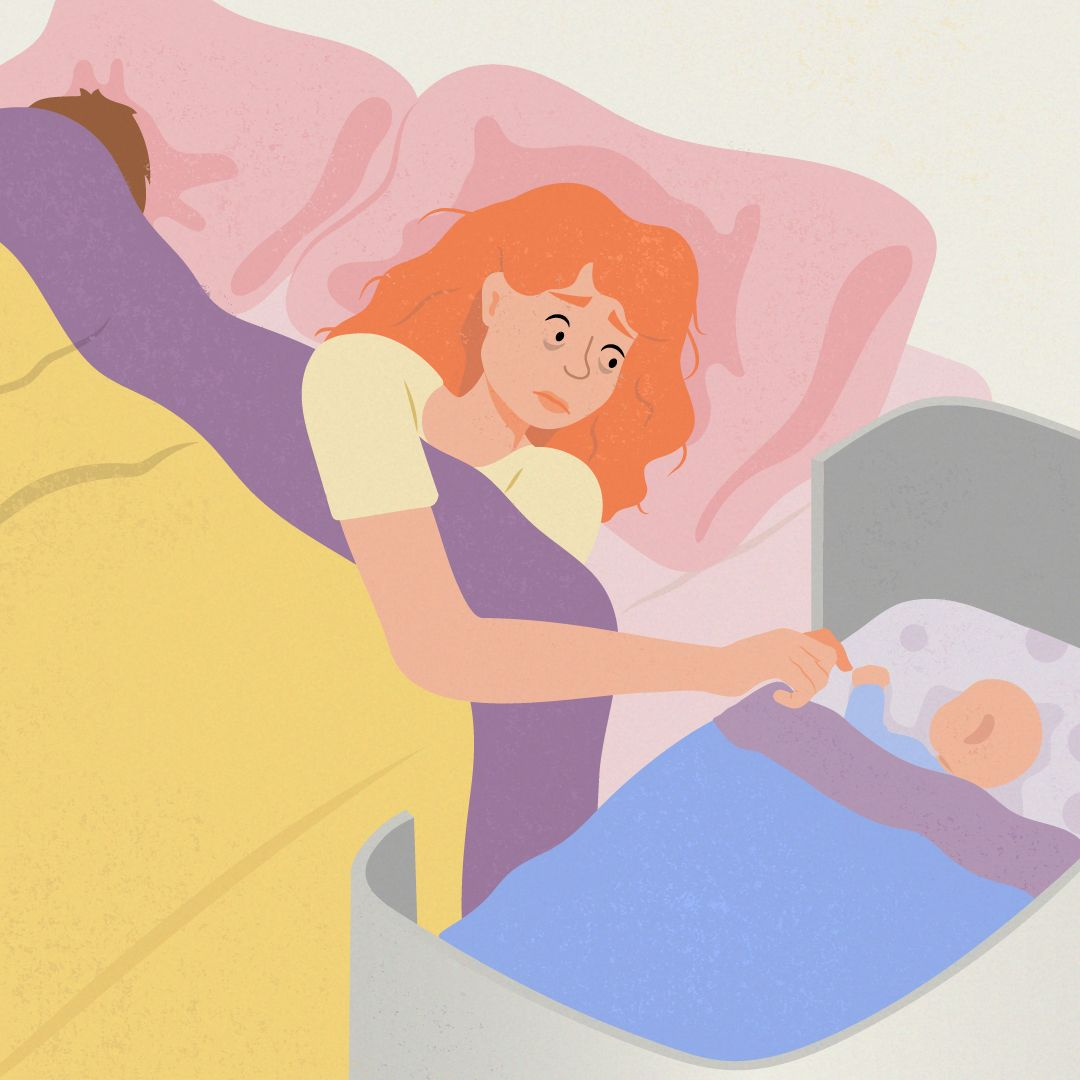

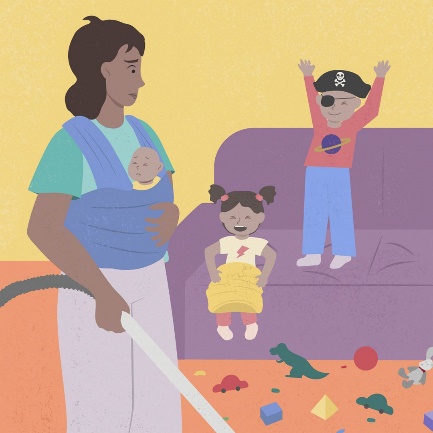


1. Set of photographs


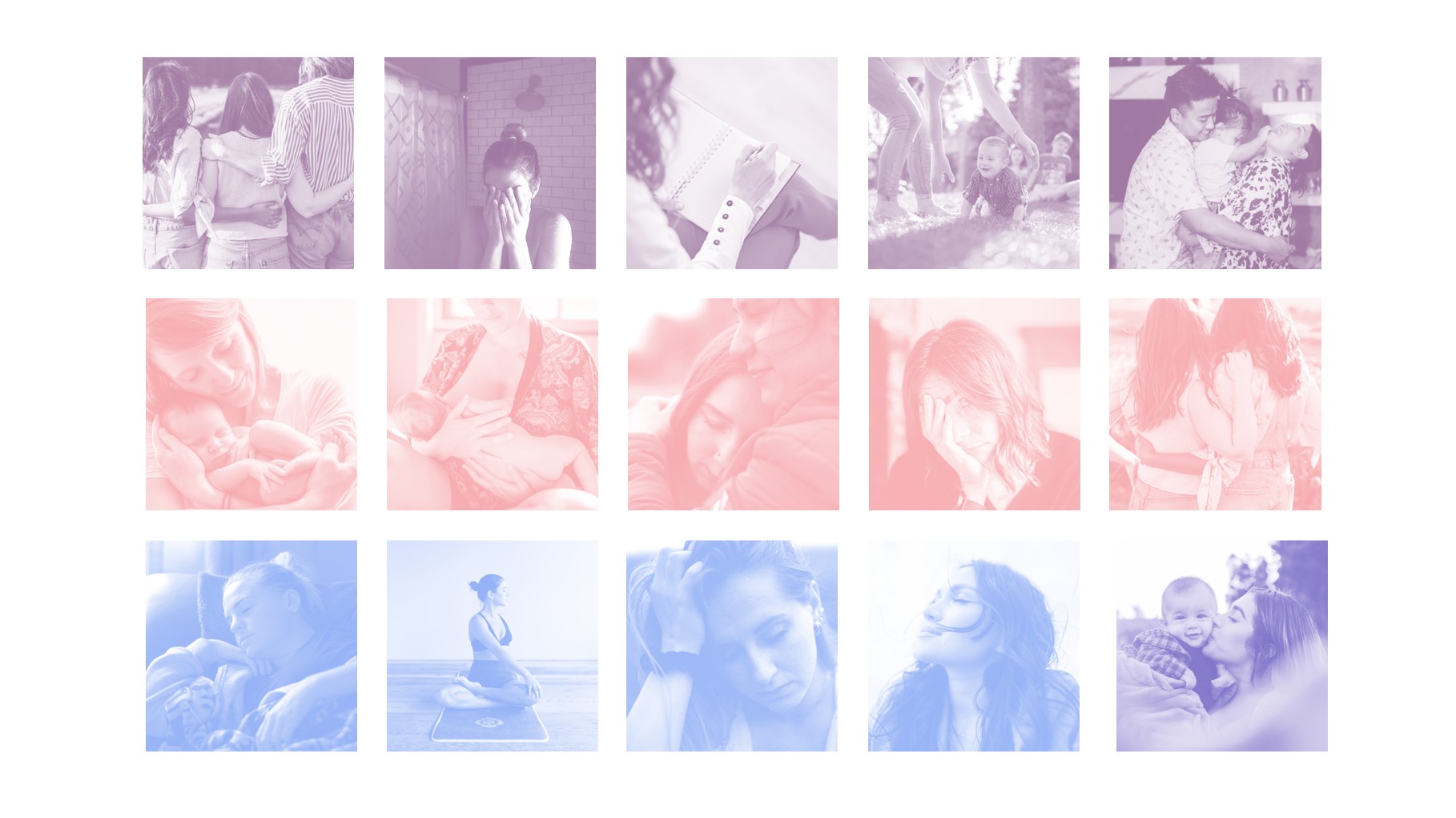


1. Overview of the Workbook section:


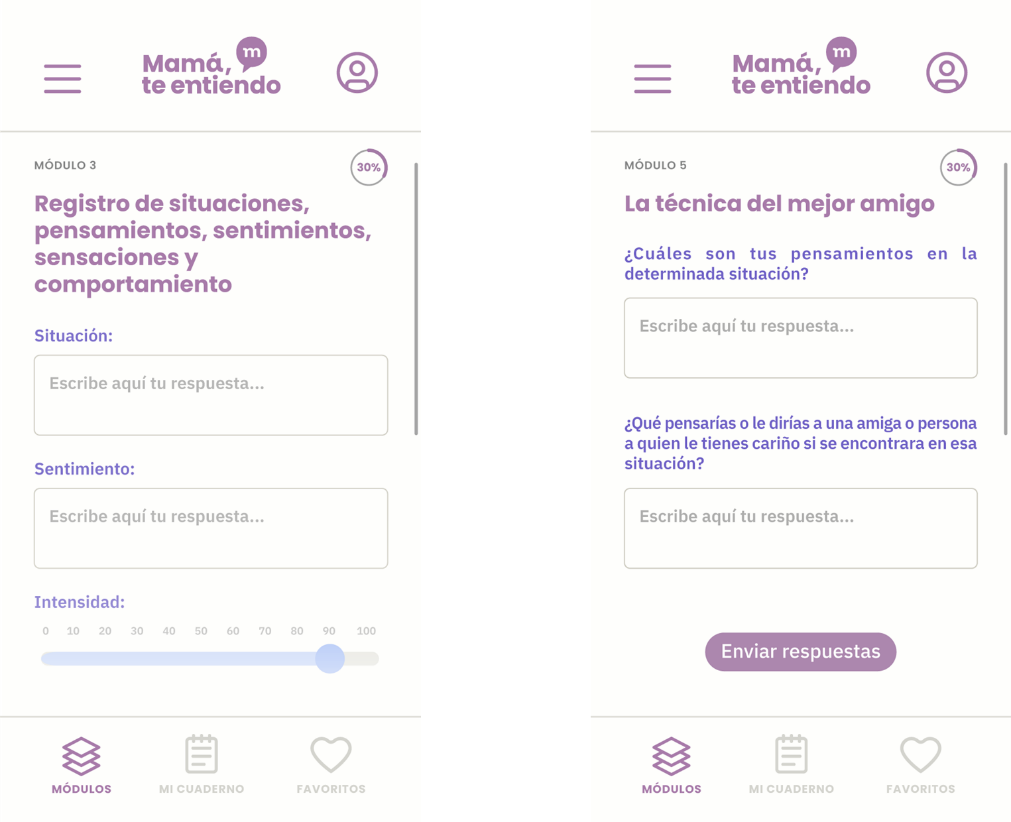

Supplement: Multimedia Appendix 2 [file formative_v8i1e56319_app2.docx]
