# Supplementary material for: Developing a Guided Web App for Postpartum Depression Symptoms: User-Centered Design Approach
Source: JMIR Form Res. 2024 Aug 19;8:e56319. doi: 10.2196/56319 (PMC11369531; doi:10.2196/56319)
Supplement: Multimedia Appendix 3 [file formative_v8i1e56319_app3.docx]

**MULTIMEDIA APPENDIX 3**

**Supplementary Tables**

Table S1. How Persuasive Systems Design Framework Elements Were Applied to the Internet-Based Intervention.

| Principle and definition according to PSD framework (Oinas-Kukkonen & Harjumaa, 2009) | | How is it applied to the internet-based intervention? |
| --- | --- | --- |
| **Primary task support** | |  |
| *Reduction* | A system that reduces complex behavior into simple tasks helps users perform the target behavior, and it may increase the benefit/cost ratio of a behavior. | The content is divided into themes displayed in modules. Exercises are also divided into small simpler steps. |
| *Tunneling* | Using the system to guide users through a process or experience provides opportunities to persuade along the way. | The intervention delivers the content in sequential modules that can only be accessed when the previous module is completed. |
| *Tailoring* | Information provided by the system will be more persuasive if it is tailored to the potential needs, interests, personality, usage context, or other factors relevant to a user group. | Women can choose if they do the exercises and if they do the extra modules.  The e-coach feedback may suggest reviewing a particular section (e.g., if an exercise from the nuclear modules shows anxiety symptoms, the coach will recommend looking at the extra module “Psychoeducation on Anxiety”). |
| *Personalization* | A system that offers personalized content or services has a greater capability for persuasion. | The e-coach feedback is personalized. The reminders and the e-coach in the exercises’ feedback speak to women with their own names. |
| *Self-monitoring* | A system that keeps track of one’s own performance or status supports the user in achieving goals. | A progress bar shows the percentage of modules read. The exercises’ progress can be seen in the Workbook section. |
| *Simulation* | Systems that provide simulations can persuade by enabling users to observe the link between cause and effect immediately. | Five women exemplify the cognitive-behavioral techniques in the app, demonstrating the effects on their mood when they use them. Additionally, the coach reinforces the cause-and-effect relationship of relevant behavior through the exercises’ feedback. |
| *Rehearsal* | A system providing means with which to rehearse a behavior can enable people to change their attitudes or behavior in the real world. | Methods or techniques come back repeatedly. |
| **Dialog support** |  |  |
| *Praise* | By offering praise, a system can make users more open to persuasion. | Women receive a congratulation or compliment when they finish a module. The e-coach praise the realization of the exercises. |
| *Rewards* | Systems that reward target behaviors may have great persuasive powers. | When they finish a module, women see an illustration representing accomplishment (e.g., a woman receiving a medal). |
| *Reminders* | If a system reminds users of their target behavior, they will more likely achieve their goals. | Not applied in this version. Including web push notifications is planned for future updates. |
| *Suggestions* | Systems offering fitting suggestions will have greater persuasive powers. | The e-coach offers personalized feedback on the exercises. |
| *Similarity* | People are more readily persuaded through systems that remind them of themselves in some meaningful way. | Women read example stories from five postpartum that cope with the most frequent problems mentioned in Chilean “real-life” consultations. |
| *Liking* | A system that is visually attractive for its users is likely to be more persuasive. | The intervention is visually designed to be attractive to the participants. It includes illustrations, icons (both designed for the intervention), and pictures. |
| *Social role* | If a system adopts a social role, users will more likely use it for persuasive purposes. | The intervention is supported by an e-coach who provides feedback on the exercises and solves doubts. |
| **System credibility support** | |  |
| *Trustworthiness* | A system that is viewed as trustworthy (truthful, fair, and unbiased) will have increased powers of persuasion. | The intervention provides information about its development and purposes, the development team, and the funding/supporting institutions. All references to the content are in a “references section”. |
| *Expertise* | A system that is viewed as incorporating expertise (knowledge, experience, and competence) will have increased powers of persuasion. | The intervention provides information about the development team and the funding/supporting institutions. The intervention presents the cognitive-behavioral techniques as “evidence-based”. |
| *Surface credibility* | People make initial assessments of the system credibility based on a firsthand inspection. | Before registering, the person must read the privacy policy and click on a “I read it” box. After registration, an “Onboarding” presents the intervention target group, its benefits, and general features. |
| *Real-world feel* | A system that highlights people or organization behind its content or services will have more credibility. | The intervention provides information about its development and purposes, the development team, and the funding/supporting institutions. Women can contact the e-coach through a messaging system embedded in the intervention if they have doubts or the research team through the corporative email created for this purpose. |
| *Authority* | A system that leverages roles of authority will have enhanced powers of persuasion. | The intervention provides information about funding/supporting institutions: the Pontificia Universidad Católica, the Millennium Institute for Depression and Personality Research, and the National Agency of Research and Development. |
| *Third-party endorsements* | Third-party endorsements, especially from well-known and respected sources, boost perceptions on system credibility. | In the future, we plan to get the intervention certified by the National Center of Health Information Systems. |
| *Verifiability.* | Credibility perceptions will be enhanced if a system makes it easy to verify the accuracy of site content via outside source. | The sources that the intervention uses can be checked. |
| **Social support** | The design principles in the social support category describe how to design the system so that it motivates users by leveraging social influence. The design principles that belong into this category, are social facilitation, social comparison, normative influence, social learning, cooperation, competition, and recognition. | Not applied. |

Table S2. Client Satisfaction Questionnaire (CSQ-8) descriptive analyses (n = 7).

| Item | Mean | SD | Score frequency (n) | | | |
| --- | --- | --- | --- | --- | --- | --- |
|  |  |  | 1 | 2 | 3 | 4 |
| Quality (item 1) | 3.71 | .48 | 0 | 0 | 2 | 5 |
| Type of service (item 2) | 3.28 | .75 | 0 | 1 | 3 | 3 |
| Met Needs (item 3) | 2.85 | 1.06 | 1 | 1 | 3 | 2 |
| Recommended (item 4) | 3.57 | .53 | 0 | 0 | 3 | 4 |
| Amount of help (item 5) | 3.71 | .48 | 0 | 0 | 2 | 5 |
| Solved problems (item 6) | 3.71 | .48 | 0 | 0 | 2 | 5 |
| Satisfied (overall) (item 7) | 3.71 | .48 | 0 | 0 | 2 | 5 |
| Come back (item 8) | 3.57 | .53 | 0 | 0 | 3 | 4 |
| Total (Sum) | 28.14 | 4.29 |  |  |  |  |

^a^CEQ-8 total scores range from 8 to 32, with higher values indicating higher satisfaction.

^b^CEQ-8 items scores range from 1 to 4.

^c^SD = Standard Deviation.

Table S3. TWente Engagement with Ehealth Technologies Scale (TWEETS) descriptive analyses (n = 7).

| Item | Mean | SD | Score frequency (n) | | | | |
| --- | --- | --- | --- | --- | --- | --- | --- |
|  |  |  | 0 | 1 | 2 | 3 | 4 |
| MTE was part of my daily routine | 2.00 | .57 | 0 | 1 | 5 | 1 | 0 |
| MTE takes me little effort to use | 3.57 | .53 | 0 | 0 | 0 | 3 | 4 |
| I was able to use MTE as often as needed to improve my mood | 3.42 | .53 | 0 | 0 | 0 | 4 | 3 |
| MTE made it easier for me to work on improving my mood | 3.28 | .75 | 0 | 0 | 1 | 3 | 3 |
| MTE motivated me to improve my mood | 3.14 | .69 | 0 | 0 | 1 | 4 | 2 |
| MTE helped me to get more insight into my thoughts and emotions | 3.28 | .95 | 0 | 0 | 2 | 1 | 4 |
| I enjoyed using MTE | 3.57 | .53 | 0 | 0 | 0 | 3 | 4 |
| I enjoyed seeing the progress I made in MTE | 3.28 | .75 | 0 | 0 | 1 | 3 | 3 |
| MTE fits me as a person | 3.28 | .75 | 0 | 0 | 1 | 3 | 3 |
| Total (Mean) | 3.20 | .55 |  |  |  |  |  |

^a^TWEETS item and total scores range from 0 to 4, with higher values indicating higher engagement.

^b^SD = Standard Deviation.
